# A Data Augmentation Methodology to Reduce the Class Imbalance in Histopathology Images

**DOI:** 10.1007/s10278-024-01018-9

**Published:** 2024-03-14

**Authors:** Rodrigo Escobar Díaz Guerrero, Lina Carvalho, Thomas Bocklitz, Juergen Popp, José Luis Oliveira

**Affiliations:** 1 BMD Software, PCI - Creative Science Park, 3830-352 Ilhavo, Portugal; 2 DETI/IEETA, University of Aveiro, 3810-193 Aveiro, Portugal; 3 Institute of Anatomical and Molecular Pathology, Faculty of Medicine, University of Coimbra, 3004-504 Coimbra, Portugal; 4 Leibniz Institute of Photonic Technology Jena, Member of Leibniz Research Alliance ‘Health Technologies’, Albert-Einstein-Straße 9, 07745 Jena, Germany; 5 Institute of Physical Chemistry and Abbe Center of Photonics (IPC), Friedrich-Schiller-University, Jena, Germany; 6 Institute of Computer Science, Faculty of Mathematics, Physics & Computer Science, Bayreuth, Germany

**Keywords:** Data imbalance, Class imbalance, Nuclei detection, Deep learning

## Abstract

Deep learning techniques have recently yielded remarkable results across various fields. However, the quality of these results depends heavily on the quality and quantity of data used during the training phase. One common issue in multi-class and multi-label classification is class imbalance, where one or several classes make up a substantial portion of the total instances. This imbalance causes the neural network to prioritize features of the majority classes during training, as their detection leads to higher scores. In the context of object detection, two types of imbalance can be identified: (1) an imbalance between the space occupied by the foreground and background and (2) an imbalance in the number of instances for each class. This paper aims to address the second type of imbalance without exacerbating the first. To achieve this, we propose a modification of the copy-paste data augmentation technique, combined with weight-balancing methods in the loss function. This strategy was specifically tailored to improve the performance in datasets with a high instance density, where instance overlap could be detrimental. To validate our methodology, we applied it to a highly unbalanced dataset focused on nuclei detection. The results show that this hybrid approach improves the classification of minority classes without significantly compromising the performance of majority classes.

**Supplementary Information:**

The online version contains supplementary material available at 10.1007/s10278-024-01018-9.

## Introduction

Deep learning techniques have recently demonstrated remarkable performance across a wide range of fields, including robotics, computer vision, and natural language processing. The effectiveness of these techniques is strongly influenced by both the quantity and quality of the training data used [1], with the accuracy of annotations playing a pivotal role in this process. However, data labeling remains a formidable challenge in numerous domains, such as histopathology, where the interpretation of vast collections of images collected daily in laboratories often necessitates highly skilled experts. Consequently, appropriately annotated datasets are scarce, expensive, and frequently with a small quantity of samples. Furthermore, histopathological images are occasionally treated as sensitive materials and are not accessible to the general public.

To address the scarcity of data, it is common practice to expand datasets artificially through data augmentation techniques, which involve creating modified versions of existing images [2]. These modifications encompass various approaches, including geometric transformations (such as flipping, cropping, rotation, translation, and noise injection), alterations in color representations (such as changes in brightness or contrast), the application of kernel filters (such as Gaussian filtering), or the addition or removal of elements within the images [3]. Alternatively, synthetic image generation techniques are employed to generate new images using methods like Generative Adversarial Networks (GAN) [4–6] or other artificial intelligence models [7, 8].

Another prevalent issue in histopathology datasets is data imbalance, where one or more classes within the dataset dominate a significant proportion of the instances. This imbalance results in the neural network assigning greater importance to the features of the majority classes during training, as their detection leads to higher scores.

Data imbalance in object detection can manifest in two forms: firstly, as an imbalance between the space occupied by the foreground and background, and secondly, as an imbalance in the number of instances per class.

In this study, we propose a methodology to address class imbalance by employing a modified version of the copy-paste (CP) data augmentation technique, coupled with weight-balancing methods integrated into the loss function. Additionally, we aim to ensure that this instance correction does not significantly compromise the balance between foreground and background spaces. To evaluate the effectiveness of our proposed methodology, we conducted experiments on a highly unbalanced histopathology dataset with a specific focus on nuclei detection.

### Related Work

Methods to mitigate class imbalance in training datasets can be categorized into three primary approaches: (a) classifier-level solutions, e.g., cost-sensitive learning [9], thresholding methods [10], or one-class classification [11]; (b) data-level solutions, such as oversampling [12, 13], or undersampling [14]; (c) hybrid [15], a combination of the two previous approaches [16, 17]. Despite the effectiveness of certain strategies in machine learning algorithms, their impact on histopathology images remains relatively underexplored. In response to this gap, Reza and Ma [18] conducted an evaluation of the effects of data imbalance on Convolutional Neural Networks (CNNs) using histopathological datasets. Their study compared the impact of oversampling and undersampling techniques in reducing the imbalance within breast cancer image datasets. Oversampling was found to be the most effective strategy in nearly every case. This same conclusion was corroborated when common images were used instead of histopathologic images [17].

In the realm of histopathology images, minority class oversampling has gained prominence, particularly in binary classification problems [19–23]. However, applying oversampling in object detection scenarios presents unique challenges, as a single image or patch may contain multiple examples, some belonging to minority classes and others to majority classes. Addressing this challenge, Hagos et al. proposed the creation of a weight matrix, where each cell carries a weighted value. This matrix is subsequently employed in the weighted dice overlap loss function to mitigate the effects of class imbalance [24]. In our approach, aimed at rectifying class imbalance, we utilize an oversampling technique combined with a weight-balancing method on the loss function.

## Methods and Materials

### Models

**Fig. 1:**
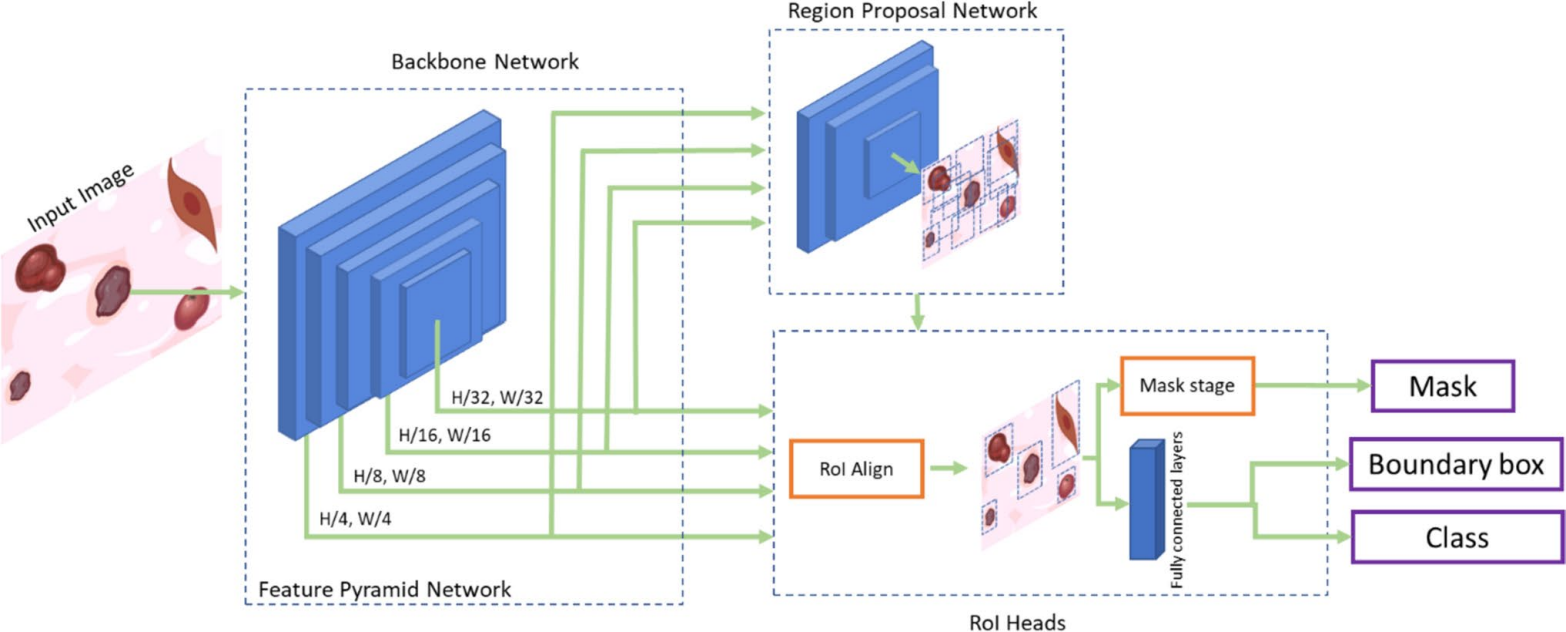
Mask RCNN summary representation

As the model, we used Mask RCNN, currently one of the most popular models for object detection and instance segmentation [25–27]. This model extends Faster RCNN by introducing an additional stage for object mask prediction, augmenting the capabilities of the Region Proposal Network (RPN) [27]. Mask RCNN comprises three primary components (Fig. 1): the Backbone network, the RPN, and the Regions of Interest (RoI) Heads. The backbone is a Feature Pyramid Network (FPN), i.e., a Convolutional Neural Network (CNN) that is used to extract the main features in multiple scales of the image. The features extracted by the FPN are used as input for the second element, the RPN. The RPN combines non-neural network functionality with a neural network to generate the RoI. This area is then refined, classified, and segmented in the RoI Heads section. For each detected instance, three outputs are produced: a segmentation mask, a bounding box, and the associated class label.

This model was implemented using Python, specifically utilizing PyTorch 1.9.0 and the open-source object detection toolbox, MMDetection [28]. To design the anchors for the RPN, we used the Pyodi tool (Python object detection insights) [29, 30].

As the backbone network, we used the FPN-based ResNeXt101-32 × 8d, known for its better performance than ResNet [31]. This is a variant of ResNet that employs a group convolution approach, where the convolutional layers are split into groups, allowing for more diverse and powerful feature extraction. The “32 × 8d’ in the name refers to the number of groups (cardinality) and the width of each group, respectively. We initialized the backbone network with pre-trained weights from the ImageNet classification task.

The number of training epochs was fine-tuned for each of the evaluated configurations, within a range spanning from 20 to 50 epochs. Stochastic Gradient Descent (SGD) was used as the optimizer in all the experiments conducted in this study. While we also explored the use of ADAM as an optimizer in our experiments, it consistently yielded inferior results across all configurations, leading us to exclude those findings from our analysis.

### Datasets

The field of computational pathology faces a significant challenge in acquiring a sufficient quantity of high-quality labels, primarily due to the substantial time and effort required from pathologists. To address this data scarcity issue in 2021, Amgad et al. developed a methodology for generating a large number of annotations through the collaborative efforts of medical students and pathologists [32]. Their dataset can be categorized into two distinct types: single-rater and multi-rater. In the single-rater category, annotations were initially created by individuals without a pathology background and subsequently refined by study coordinators, all of whom were supervised by a pathologist. In the multi-rater category, seven pathologists independently generated annotations for the same set of images, and these annotations were later consolidated into a single dataset.

For the training and validation phases, we employed the Corrected Single-Rater Dataset (CSRD), and for the test phase, we turned to the Inferred P-truth from the Evaluation Multi-Rater Dataset (IPEMRD). The CSRD consists of 1744 Fields of Views (FOVs) containing over 59,000 annotated nuclei derived from breast cancer images. Each nucleus is categorized into one of 13 distinct classes, namely: ‘tumor,’ ‘fibroblast,’ ‘lymphocyte,’ ‘plasma_cell,’ ‘macrophage,’ ‘mitotic_figure,’ ‘vascular_endothelium,’ ‘myoepithelium,’ ‘apoptotic_body,’ ‘neutrophil,’ ‘ductal_epithelium,’ ‘eosinophil,’ and ‘unlabeled.’ The second dataset, IPEMRD, comprises 53 FOVs with more than 1370 annotations sourced from breast cancer images. It shares the same classes and file format as the CSRD.

**Fig. 2:**
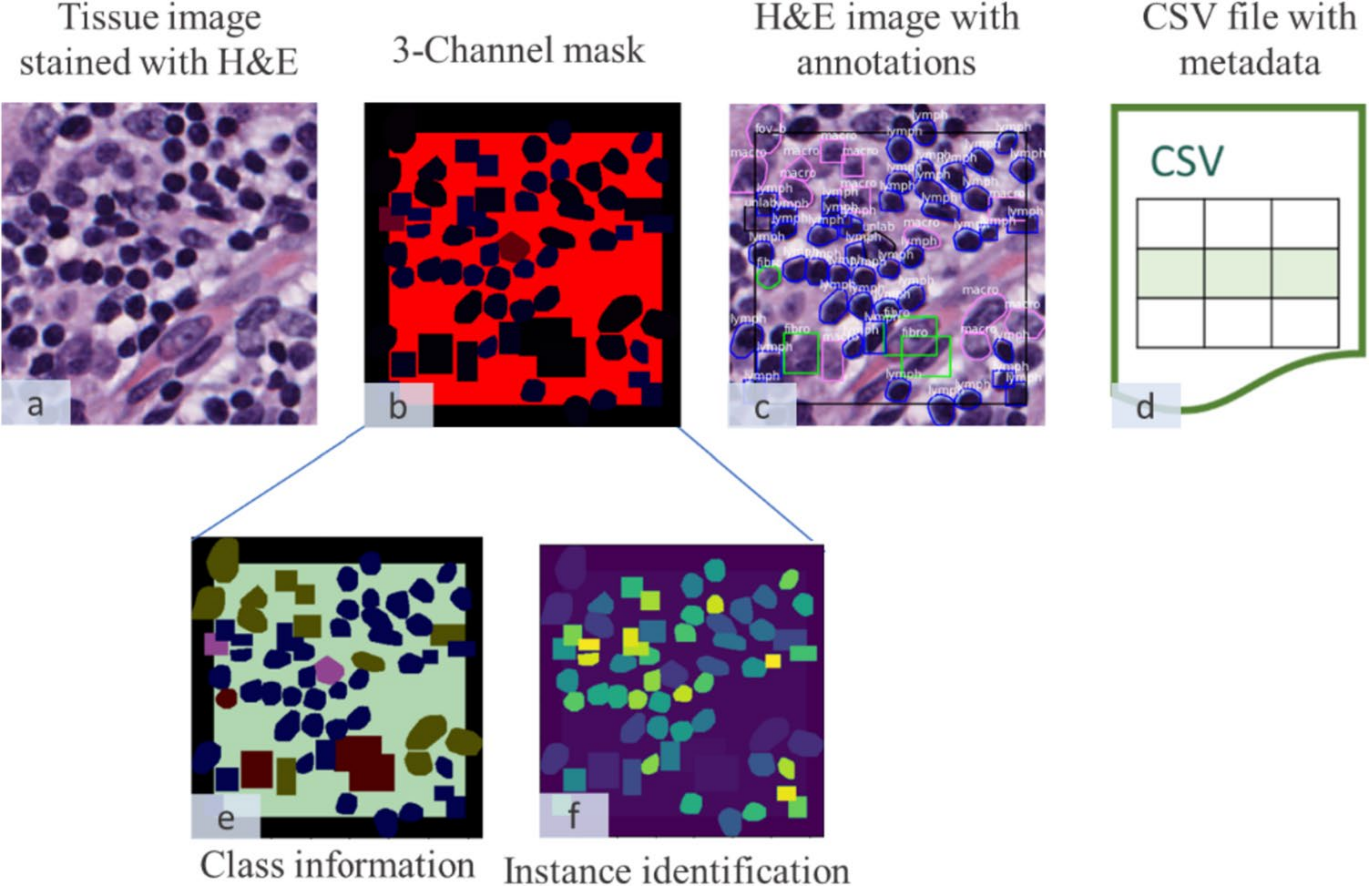
Visual representation of dataset components. **a** Tissue image (H&E staining). **b** 3-Channel mask. **c** H&E image with annotations. **d** CSV metadata. **e** Red channel: class information. **f** Blue channel: instance identification. The **e** and **f** have been modified for better visualization; they are originally intensity maps in grayscale

For each FOV, the datasets provide four files: a tissue image (Fig. 2a) stained with hematoxylin and eosin (H&E), a 3-channel mask (Fig. 2b), an H&E image with annotations overlaid (Fig. 2c), and a CSV file containing metadata for each annotation (Fig. 2d).

The 3-channel mask contains valuable information distributed across its channels, with each channel functioning as an intensity map. In the red channel (Fig. 2e), each class is represented by a distinct intensity value. For instance, if we consider the ‘tumor’ class, all the nuclei belonging to this class share the same intensity value, such as a pixel value of 3. On the other hand, the blue channel (Fig. 2f) serves as an instance map, assigning a unique pixel value to each individual nucleus. This information allows us to determine the class associated with every pixel in the image and distinguish between individual instances effectively. In our experiments, we opted to use this information rather than relying on the data provided in the CSV file. The CSV file lacked consistency in how instance data was presented, and there were cases of duplicate information. Table 1 shows the distribution for each instance after the 3-channel mask has been used for its identification.

**Table 1:** Instance distribution inside NuCLS datasets

**LABEL**	**INSTANCES IN CSRD**	**INSTANCES IN IPEMRD**
TUMOR	21,088	510
LYMPHOCYTE	13,575	207
FIBROBLAST	8639	230
UNLABELED	7518	150
PLASMA_CELL	5557	161
MACROPHAGE	1353	42
VASCULAR_ENDOTHELIUM	514	48
DUCTAL_EPITHELIUM	498	0
APOPTOTIC_BODY	391	13
MITOTIC_FIGURE	229	5
MYOEPITHELIUM	55	0
NEUTROPHIL	45	6
EOSINOPHIL	3	0

### Evaluation Metrics

#### Mean Average Precision

One of the key metrics used to validate our results is Mean Average Precision (mAP), a commonly used metric for assessing object detection and instance segmentation methods. This metric provides a single number within the range of 0 to 1, allowing us to evaluate overall performance effectively. mAP is defined as the mean area under the curve of the interpolated precision-recall curve for all classes [33].

**Fig. 3:**
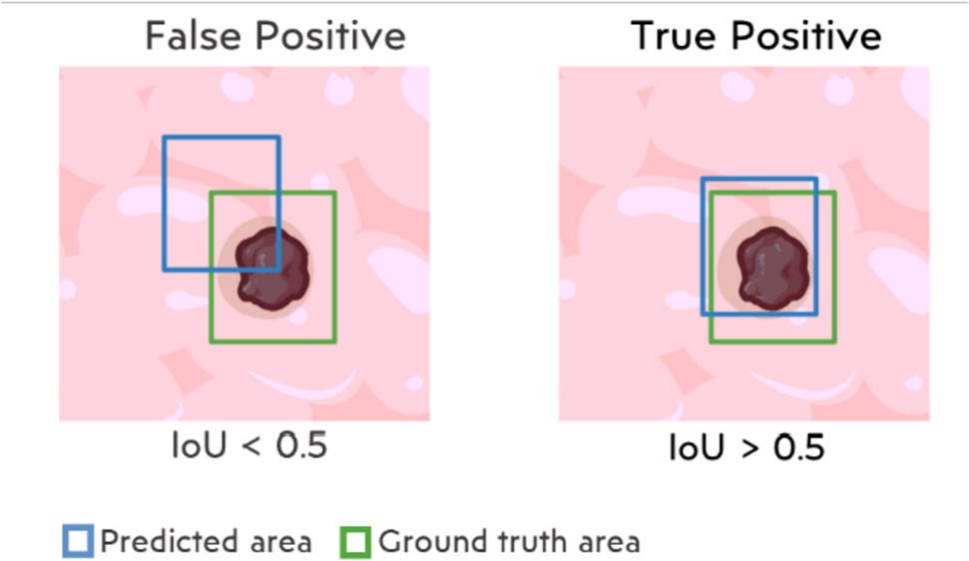
Visual representation of outcomes with IoU threshold set to 0.5. Blue rectangles: predicted areas. Green rectangles: ground truth areas

To understand how mAP is calculated, it is essential to define the parameters involved in its computation. The Intersection over Union (IoU) plays a crucial role in determining how well the predicted region of an object matches the ground truth region. It is defined as the area of overlap between the predicted and ground truth regions divided by the area of their union. A validation threshold is specified to determine whether a detection is correct or not. Any IoU value exceeding this threshold is considered a true positive (TP), while values below it are considered false positives (FP). In our experiments, we used a threshold of 0.5 to evaluate the results. An illustrative example of TP and FP with an IoU threshold of 0.5 is provided in Fig. 3.

Once the TP and FP values are obtained, it is possible to calculate the precision and recall (also called sensitivity). Precision is a measure of the accuracy of positive predictions, while recall gauges the completeness of positive predictions.

The precision and recall for each instance within a class can be represented in a plot called the *precision-recall curve*. The area under this curve is referred to as Average Precision (AP) and is defined as:

$$AP@\alpha = {\int }_{0}^{1}p\left(r\right) dr$$

where $\alpha$ is the IoU threshold and $p\left(r\right)$ is the precision-recall curve.

To generate the mAP, the AP of each class is calculated through different thresholds (in our experiments only 0.5 is used). Finally, the average of all AP is used to produce the mAP and is defined as:

$$mAP= \frac{1}{n}\sum_{k=1}^{k=n}{AP}_{k}$$

where ${AP}_{k}$ is the AP class of class *k* and *n* is the number of classes.

#### Balanced Accuracy

Balanced accuracy is a measure of classification model performance that considers the average of sensitivity and specificity, making it robust to class imbalance and suitable for evaluating binary or multi-class classification tasks.

### Dataset Configurations

**Fig. 4:**
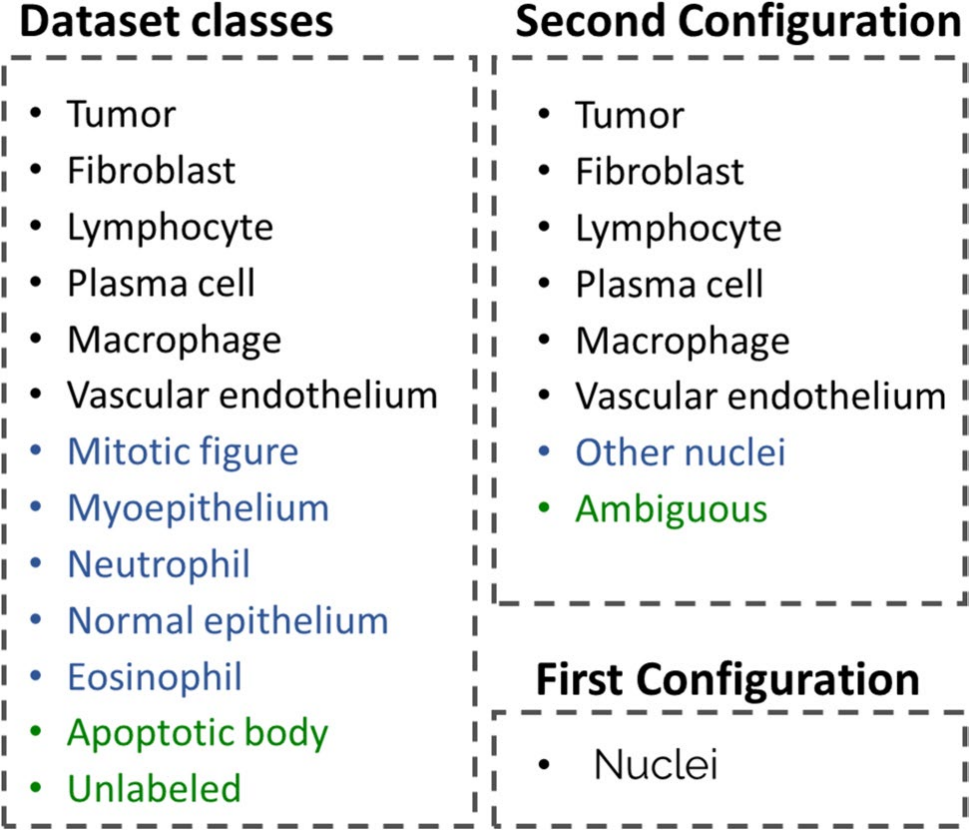
Diagram of different dataset configurations. The first configuration groups all classes from the dataset, and the second configuration groups minority classes

Two different configurations of the datasets were evaluated (Fig. 4). Each configuration serves a specific purpose in assessing our methodologies. In the first configuration, we amalgamated all classes into a single category labeled “Nuclei.” This setup allows us to evaluate nuclei detection independently, disregarding the classification aspect. Weight balancing in the loss function is not applicable in this configuration due to the presence of only one class. In the second configuration, we grouped certain classes together. Specifically, we combined the classes *Mitotic figure*, *Myoepithelium*, *Neutrophil*, *Normal epithelium*, and *Eosinophil* into a single class labeled “Other nuclei.” Similarly, the classes Apoptotic body and Unlabeled were grouped under the class “Ambiguous.” It was chosen to group the classes instead of using the original classes due to the small number of instances of some classes, e.g., eosinophil has only 3 samples, while tumor has more than 21,000.

## Proposed Method

Data imbalance reduction techniques can be categorized into three primary groups: (1) data-level methods, (2) classifier-level methods, and (3) hybrid methods. Data-level methods involve increasing the number of samples for minority classes or reducing the instances of majority classes. Classifier-level involves adapting algorithms to effectively handle imbalanced datasets. Hybrid methods combine elements from both of the aforementioned groups [16].

In this paper, we propose a hybrid method that combines two techniques: augmenting the number of instances using a modified copy-paste (MCP) method and applying rescaled class weights in the loss function.

### Copy-Paste Modification

In recent years, the copy-paste (CP) method has demonstrated effectiveness in instance segmentation and object detection [34, 35]. This method involves randomly copying and pasting instance samples into images within the dataset. Due to the randomness of pasting, there is a high probability of overlap, particularly when images have a high instance density, as is often the case with tissue nuclei. This overlap can lead to complete occlusion, especially when dealing with instances of similar sizes, and may introduce an imbalance between foreground and background, making object identification more challenging.

To address these issues, we propose a modification to the CP algorithm that avoids the overlap between instances and reduces the imbalance between classes. Before applying the CP method, we incorporate a preprocessing stage where we perform color normalization on the entire dataset using the Reinhard algorithm [36, 37]. Color normalization, as suggested by several authors [37–39], enhances the performance of deep learning techniques on images stained with hematoxylin and eosin dyes.

Additionally, we transform all annotations in the dataset into the COCO (Common Object in Context) format, which is one of the most widely used formats for object detection and segmentation datasets. Created for the Microsoft COCO dataset, it has now been adopted for various other data collections [40–42]. This JSON-based format stores crucial information, including image information, object annotations, and different categories or classes, simplifying instance retrieval and dataset management.

**Fig. 5:**
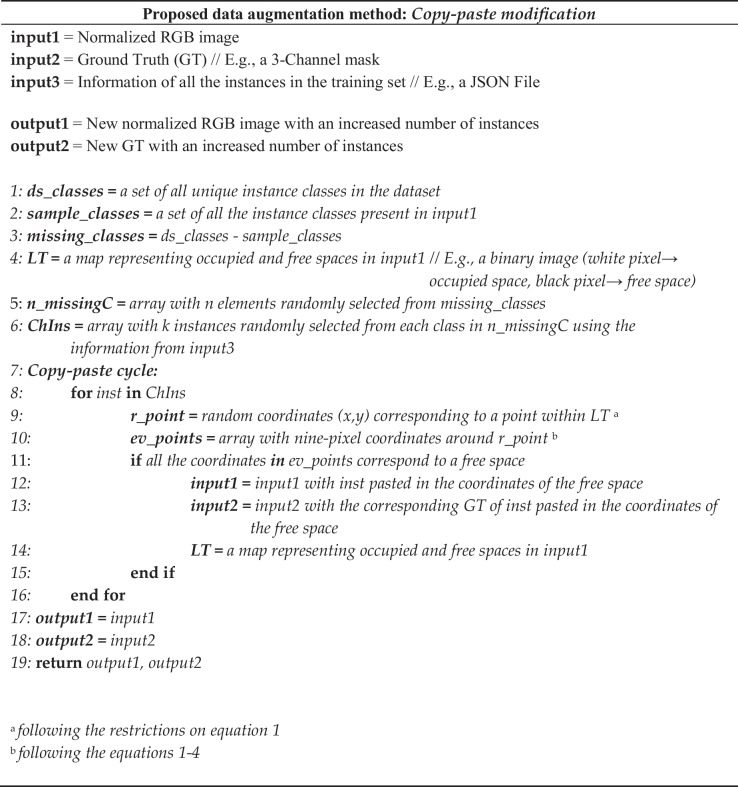
Pseudocode of our copy-paste modification

**Fig. 6:**
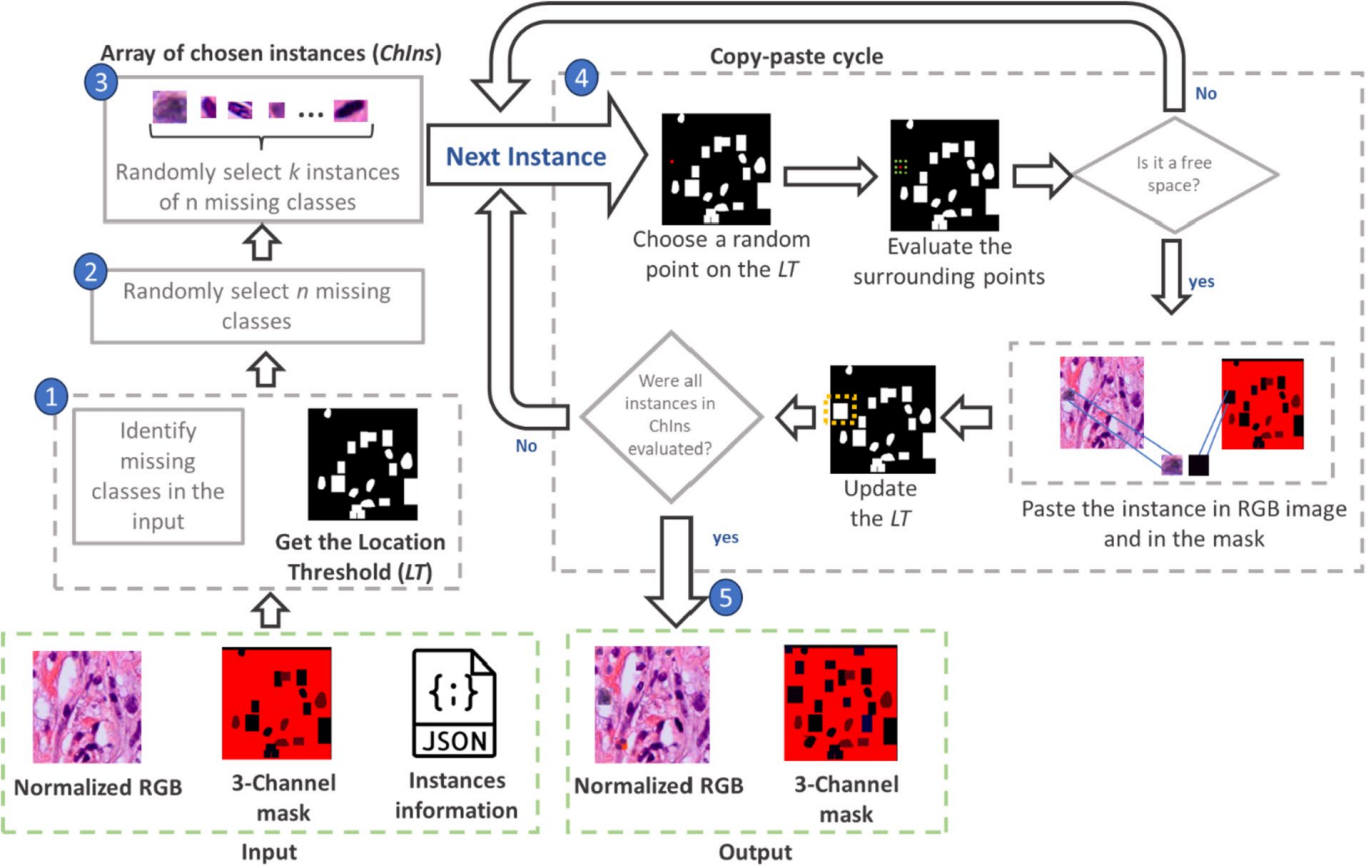
Summary of the steps involved in our proposed data augmentation approach for images with high instance density and high class imbalance

After converting the data into the COCO format and normalizing the image colors, it is possible to start our proposed data augmentation method. In Fig. 5, we provide a detailed pseudocode outlining the main steps of our proposal, and in Fig. 6, we present a visual diagram summarizing the key steps.

We can summarize the process in the following 5 steps (Fig. 6):

1. For each image, identify the missing classes and create a location threshold (LT). LT is a reference map that shows the spaces occupied within the image by the instances. We generate a binary mask where white pixels correspond to instances, and black pixels represent spaces without instances (i.e., areas without nuclei).
2. Randomly select *n* elements from the missing classes.
3. From each of these *n* missing elements, randomly select k samples from the whole training dataset. This selection results in an array that we call ChIns (chosen instances).
4. Each element in ChIns enters the copy-paste cycle, where we determine whether a selected location is available for pasting the instance.
5. Finally, the copy-paste is executed, returning the new image with an augmented number of instances and its corresponding ground truth (GT).

The copy-paste cycle proceeds as follows:

Begin by extracting the height and width information of the first instance within the ChIns array. Then, choose a random point ${p}_{0}$*(x,y)* within *LT(h,w)* following the next conditions:

1 $${p}_{0}\left(x,{\text{y}}\right)=\left\{\begin{array}{c}x=rx, if \left(\frac{{w}_{ins}}{2}\right)<rx<\left({w}_{LT}-\left(\frac{{w}_{ins}}{2}\right)\right)\\ y=ry, if \left(\frac{{h}_{ins}}{2}\right)<ry< \left({h}_{LT}-\left(\frac{{h}_{ins}}{2}\right)\right)\end{array}\right.$$

Here, *rx* and *ry* are random *x* and *y* axes, *w*_*LT*_ and *h*_*LT*_ represent the width and height of *LT*, and *w*_*ins*_ and *h*_*ins*_ are the width and height of the instance.

Next, eight new points *p*_*n*_ around *p*_*0*_ are created using the following coordinates:

2 $${p}_{n}\left(x,y\right)=\left({p}_{{0}_{x}}+{k}_{x},{p}_{{0}_{y}}+ {k}_{y}\right)$$

where:

3 $${k}_{x}= \left\{-\left(\frac{{w}_{ins}}{2}\right), 0, \left(\frac{{w}_{ins}}{2}\right)\right\}$$

and

4 $${k}_{y}= \left\{-\left(\frac{{h}_{ins}}{2}\right), 0, \left(\frac{{h}_{ins}}{2}\right)\right\}$$

Except when *k*_*x*_ = *k*_*y*_ = 0.

The total of the nine points is used to evaluate if that location is free, i.e., if all the pixels in LT, according to the coordinates of those points, are black, then it is considered a free space. In this case, the instance is pasted into the normalized image, along with its corresponding GT. *LT* is updated, and the copy-paste cycle is repeated with the next instance within *ChIns*. If any of the nine points corresponds to a white pixel, the space is considered occupied, and to prevent overlapping, this instance is not pasted. Once all the instances in ChIns have completed the copy-paste cycle, the addition of instances in that image is completed. The process is then repeated with all images within the training set.

It is worth noting that when applying this methodology to other datasets, several considerations should be taken into account:

- *Instance size variations*: If instances significantly differ in size, an alternative evaluation method should be proposed, as the nine-point evaluation works best when instances have similar sizes. Significant variations may lead to occlusions.
- *Customization*: The parameters, such as the number of classes (*n*) and the number of instances (*k*), should be adjusted to suit the characteristics of each dataset.

### Weight Balancing in the Loss Function

Weight balancing in the loss function is a widely adopted strategy to address imbalanced datasets. This technique involves assigning different weights to each class to ensure that minority classes receive more attention during training than elements from the majority classes. The weight for each class, denoted as *w*, can be calculated using the following equation:

5 $$w= \frac{Number\;of\;instances}{\left(Number\;of\;classes * \alpha \right)}$$

where $\alpha$ is an array with the number of occurrences for each class.

Different loss functions are implemented in the Mask RCNN model, but in our approach, only the weights of the loss function related to object classification were modified. For this task, we used a Cross-Entropy loss function which is defined as:

6 $${L}_{CE}=-\sum_{c=1}^{M}{w}_{c}\mathit{log}\left({p}_{o,c}\right){y}_{o,c}$$

where $M$ is the number of classes, *w* is the corresponding weight for each class, $p$ is the predicted probability observation $o$ is of class $c$, and $y$ is a binary indicator, which is one when class label $c$ is the correct classification for observation *o*.

It is important to note that applying weight balancing solely to the loss function enhances the identification of minority classes. However, as we will demonstrate in the “Results and Discussion” section, this approach may lead to a decrease in performance for other evaluation metrics.

## Experiment Setup

All experiments were conducted on a computer equipped with an AMD Ryzen Threadripper 3960 × processor with 24 cores, 128 GB of RAM, and two GeForce 3090 GPUs, each with 24 GB of VRAM.

**Fig. 7:**
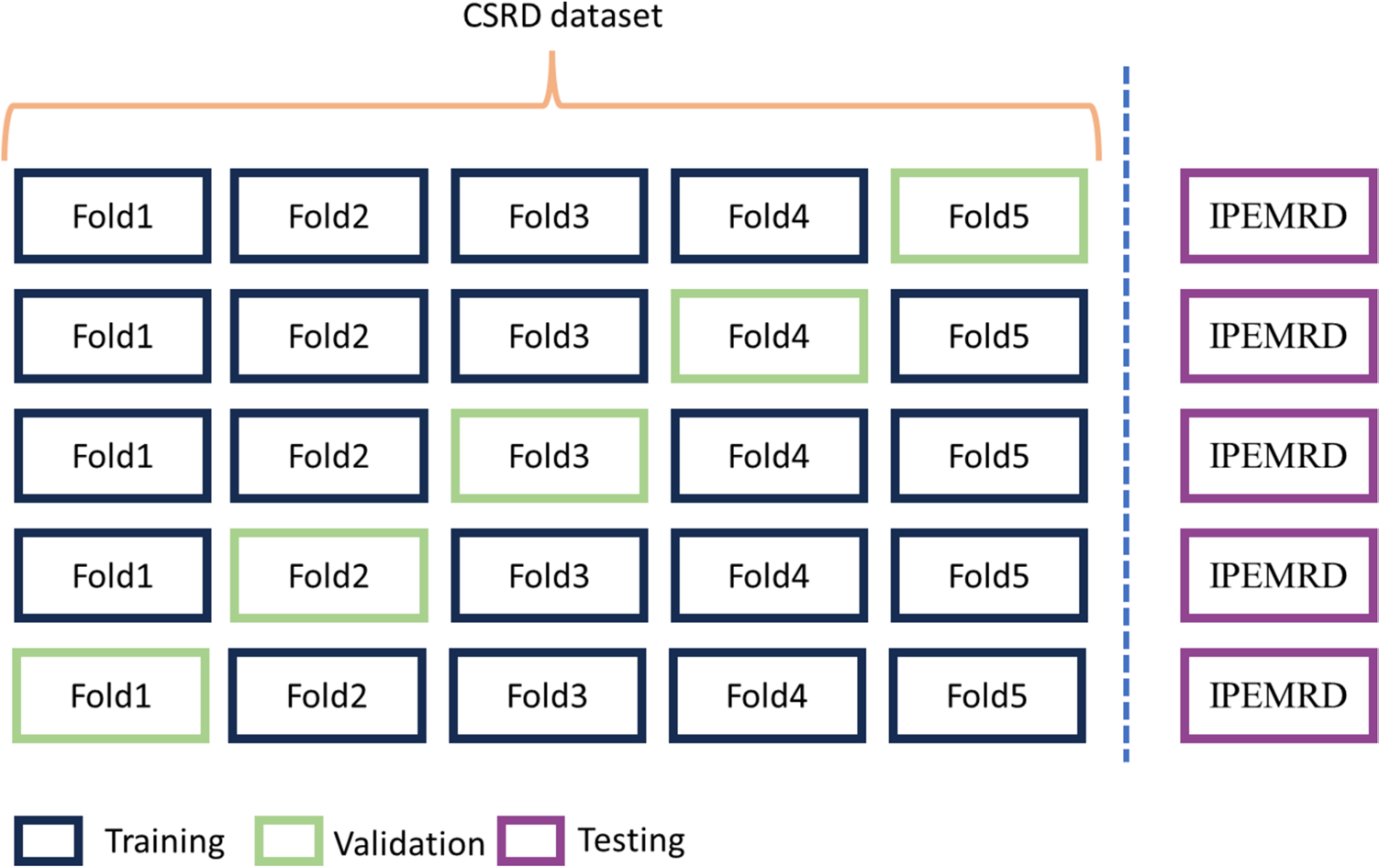
Experiment setup following 5 cross-validation data with a train-validate-test split

We implemented a fivefold cross-validation with a train-validate-test split (Fig. 7) to ensure a rigorous evaluation of our model. During the training and validation phases, we exclusively utilized the CSRD dataset, while the IPEMRD dataset served as our test set.

It is essential to note that we applied data augmentation exclusively to the training set, ensuring that the validation and test sets remained untouched by any augmentation techniques. For a comprehensive overview of the instance distribution within each dataset configuration, refer to Table 2. Furthermore, this table includes the instance count both before and after implementing our CP modification. These values show a significant increase in minority class instances compared to the majority class instances, leading to a more balanced dataset.

As no augmentation should be applied to the test set, it retains its original instances. The absence of detected elements from classes with few instances significantly influences the results. For instance, as shown in Table 2, in the test set, the class “Other Nuclei” has only 11 instances, highlighting the challenges in correctly classifying minority classes.

**Table 2:**
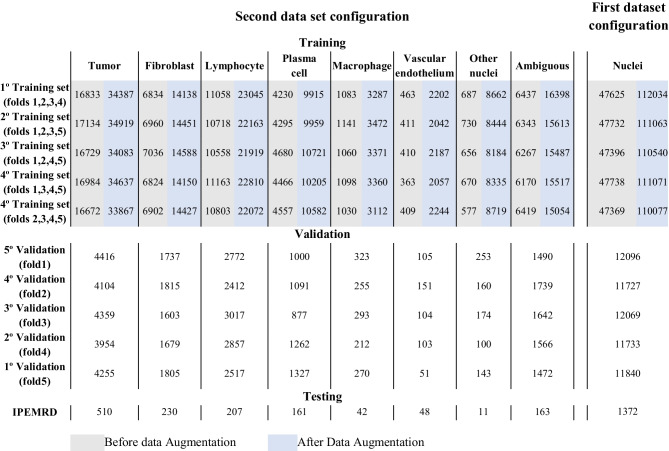
Instance distribution in each dataset configuration

The following experiments were carried out in each dataset configuration:

- *Without data augmentation (WDA):* Original training set without any data augmentation and using the Cross-Entropy loss function, with all classes assigned equal weights (weight = 1).
- *Basic data augmentation (BDA)*: Data augmentation by a random flip (horizontal and vertical) with a ratio of 0.5.
- *Modified copy-paste data augmentation (MCP)*: Using the proposed CP method to reduce the imbalance in a dataset with images that have a high density of instances.
- *Modified copy-paste plus a basic data augmentation (MCP* + *BDA):* A combination of two different methods of data augmentation.
- *Changing Cross-Entropy Loss Function to Focal Loss (FL):* Focal loss is a loss function used in several studies to address class imbalance in tasks like object detection. The gamma value used was 2.0 and the alpha value was 0.25, as proposed by Lin et al. [43].
- *Weighted Cross-Entropy Loss Function (WCEL):* Modifying the weights to match the number of classes in the second configuration. This experiment was not performed in the first configuration, as it contains only one class, resulting in the same outcome as WDA.
- *Weighted Cross-Entropy plus modified copy-paste data augmentation (WCEL* + *MCP):* A combination of copy-paste data augmentation proposed and weighted Cross-Entropy loss function. As with WCEL, this experiment was not conducted in the first configuration due to its single-class nature.

This comprehensive set of experiments allowed us to assess the impact of various strategies on model performance, as elaborated in the “Results and Discussion” section.

## Results and Discussion

### First Dataset Configuration

Object detection comprises two fundamental tasks: object localization and object classification. It is crucial to evaluate the performance of both tasks. Accordingly, our evaluation commences with an examination of the outcomes of our first configuration, which does not encompass nuclei classes. Our primary objective here is the validation of nuclei localization accuracy.

**Fig. 8:**
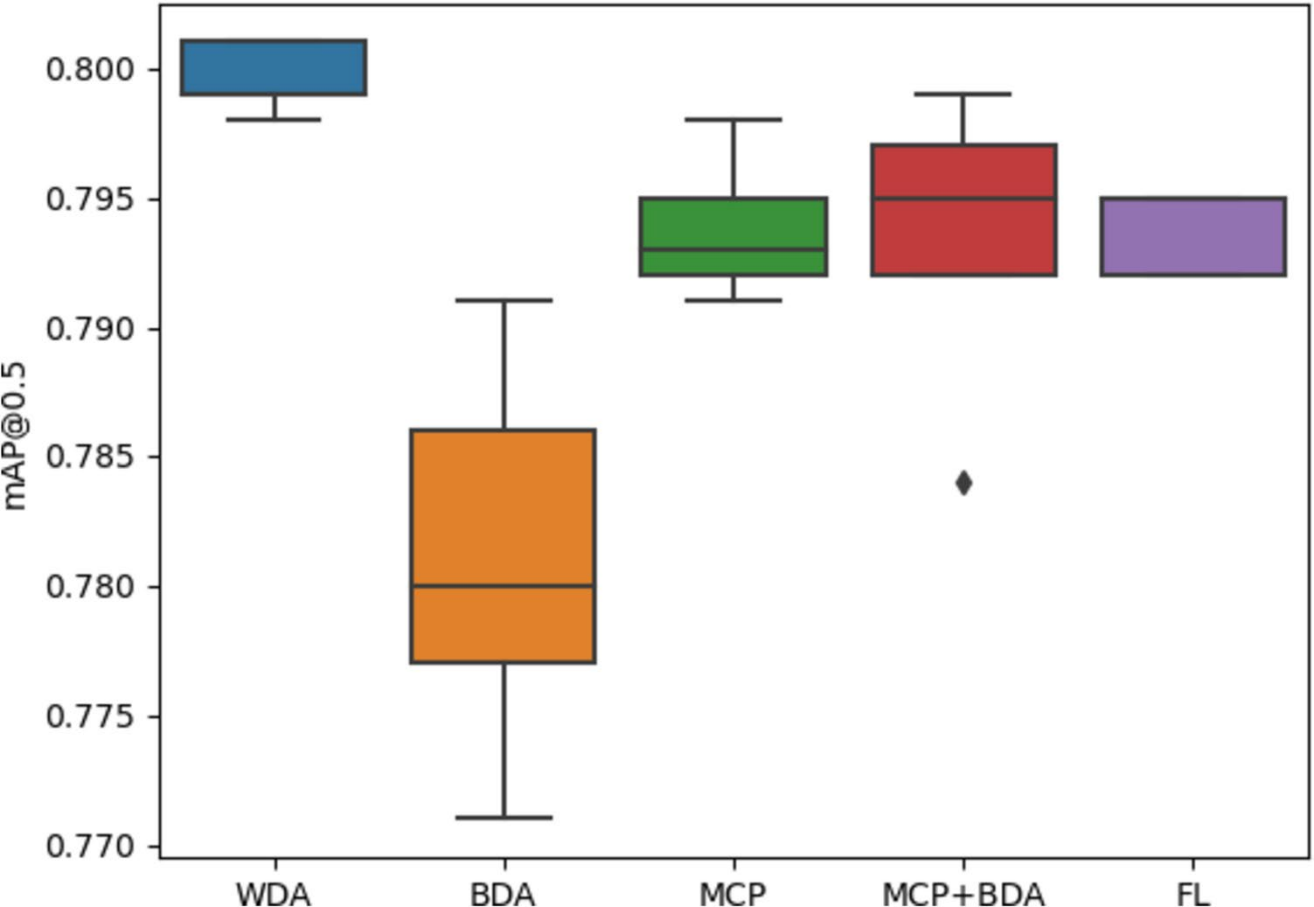
Boxplot of mAP@0.5

To assess the localization accuracy, we initiate our analysis by scrutinizing the mean Average Precision at IoU (Intersection over Union) threshold 0.5, denoted as mAP@0.5, across all experiments. From Fig. 8, we observe that the mAP@0.5 scores in all experiments are consistently close to 0.8. This consistency underscores the excellent precision and recall for nuclei localization, irrespective of the methods employed to address class imbalance. In essence, these methods have no significant impact on the accurate localization of nuclei.

To validate this assumption, we conducted a Kruskal–Wallis test [44], a non-parametric method, to discern differences among independent groups. This test, relying on ranked data to compute an *H*-statistic, evaluates variations between groups. Surprisingly, the test yielded an *H*-statistic of 16.9751, corresponding *p*-value of 0.0019, indicating significant differences among the groups.

Following the Kruskal–Wallis test, we conducted a Dunn’s test [45] using the Bonferroni [46] correction to explore specific disparities among these groups. The resulting matrix of *p*-values (Table 3) from Dunn’s test highlights a significant difference between the BDA and WDA methods; the rest of the methods as we expected does not have a significant difference to locate nuclei. Values of 1 along the diagonal signify comparisons of the same groups, indicating no significant differences. Conversely, lower *p*-values off the diagonal signify notable distinctions between specific pairs of groups. We are using a significance level of 0.05, where any *p*-value below this threshold is deemed statistically significant.

**Table 3:** Matrix of *p*-values derived from Dunn’s test comparing different methods: without data augmentation (WDA), basic data augmentation (BDA), modified copy-paste data augmentation (MCP), modified copy-paste plus basic data augmentation (MCP + BDA). The values represent pairwise comparisons, *p*-values below a significance level of 0.05 are highlighted in bold

	**WDA**	**BDA**	**MCP**	**MCP + BDA**	**FL**
**WDA**	1	**0.0003**	0.3048	0.5689	0.3582
**BDA**	**0.0003**	1	0.5148	0.2730	0.4418
**MCP**	0.3048	0.5148	1	1	1
**MCP + BDA**	0.5689	0.2730	1	1	1
**FL**	0.3582	0.4418	1	1	1

**Fig. 9:**
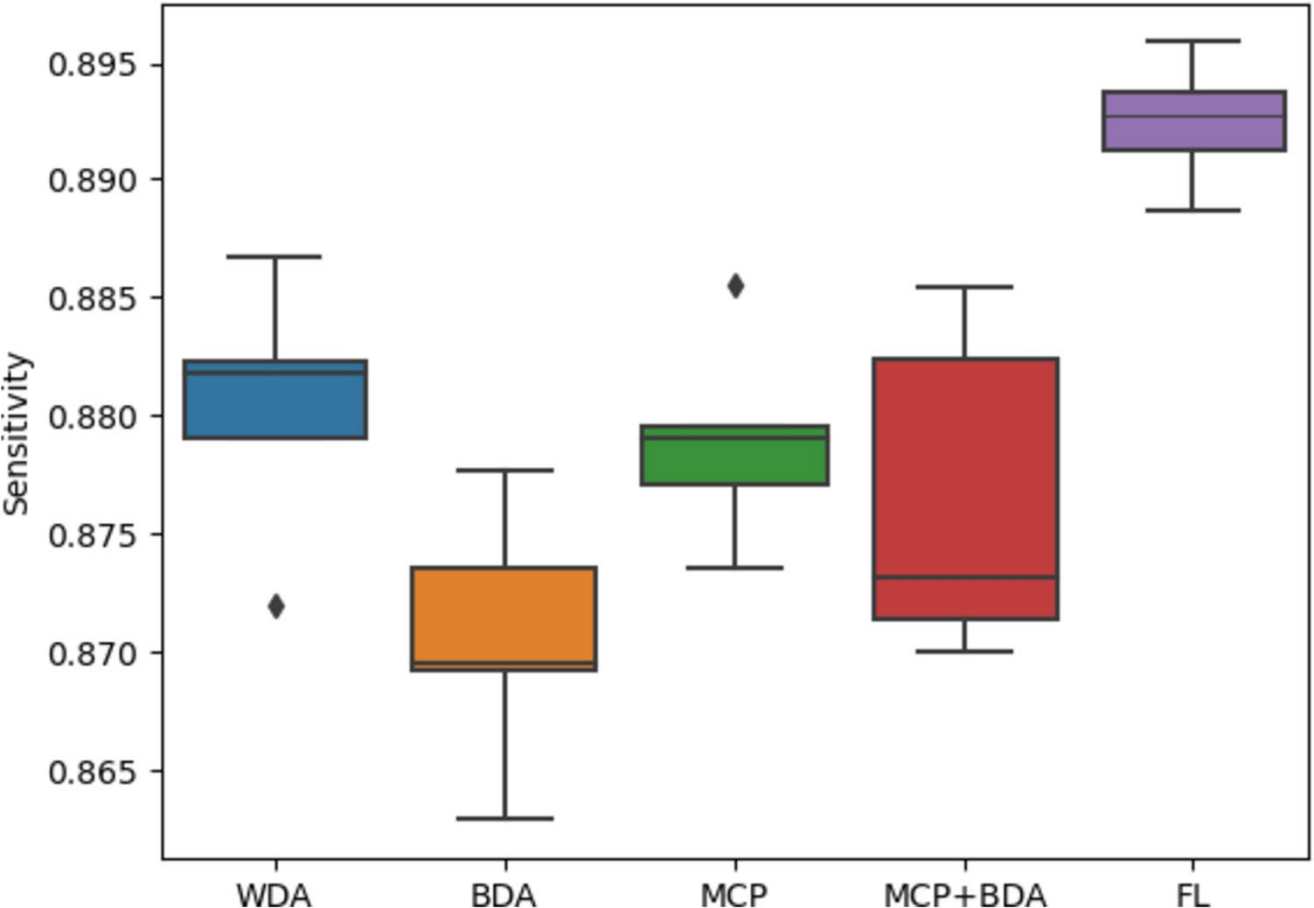
Boxplot of sensitivity

Furthermore, our evaluation extended to the sensitivity metric, which in this context reflects the ability to accurately detect nuclei. The sensitivity range observed in Fig. 9, spanning from 0.862 to 0.895, implies that a segment of nuclei instances, approximately 11 to 14%, remains undetected by all the methods employed.

### Second Dataset Configuration

Having established that the detection rate for nuclei peaks at 89.5%, signifying that approximately 144 nuclei remain undetected in the test set, a pertinent question arises: to which class do these undetected nuclei belong, and how well are they classified once detected? To address these queries, we turn our attention to the second configuration.

**Fig. 10:**
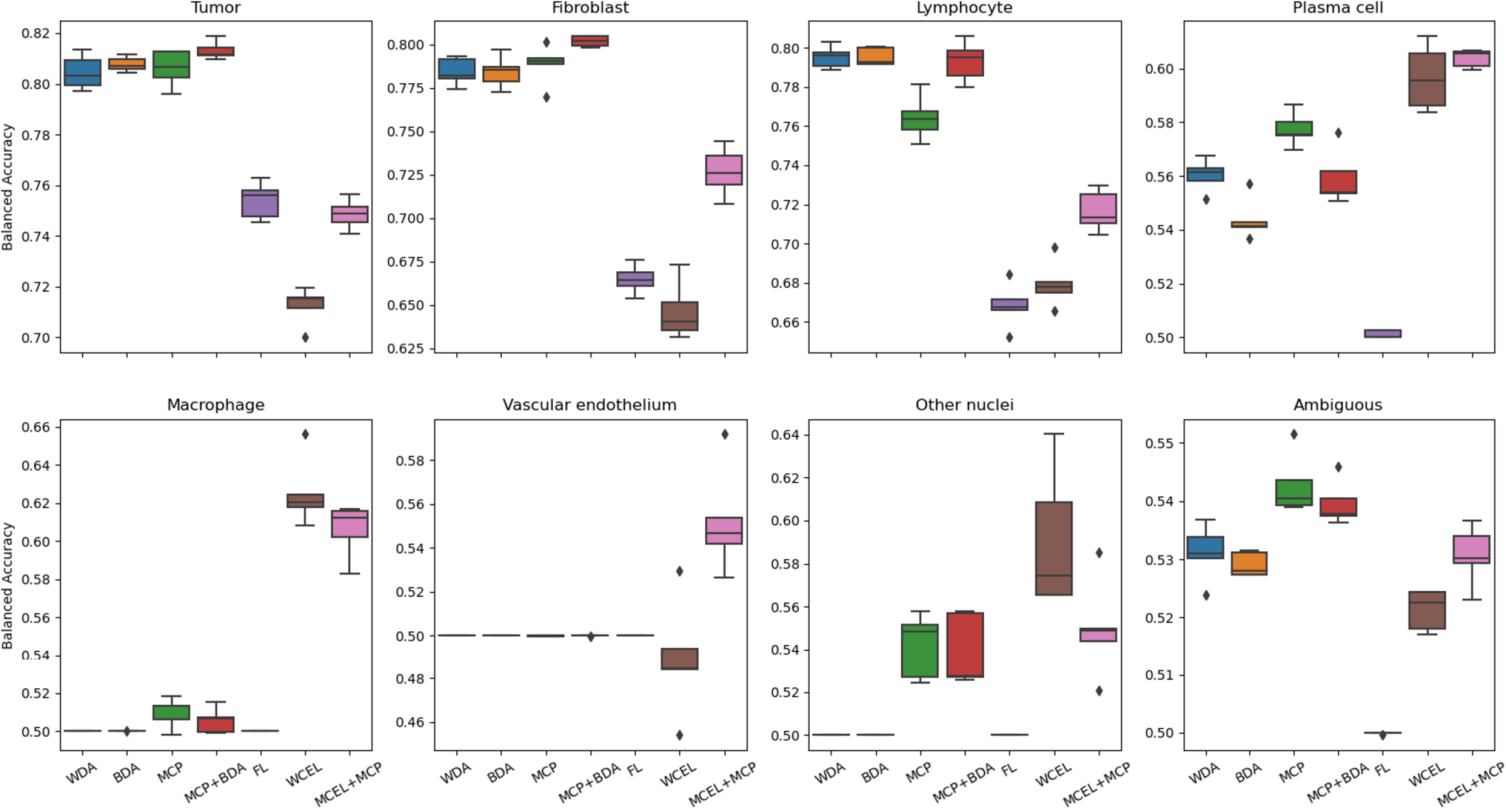
Class-balanced accuracy considering background label

**Fig. 11:**
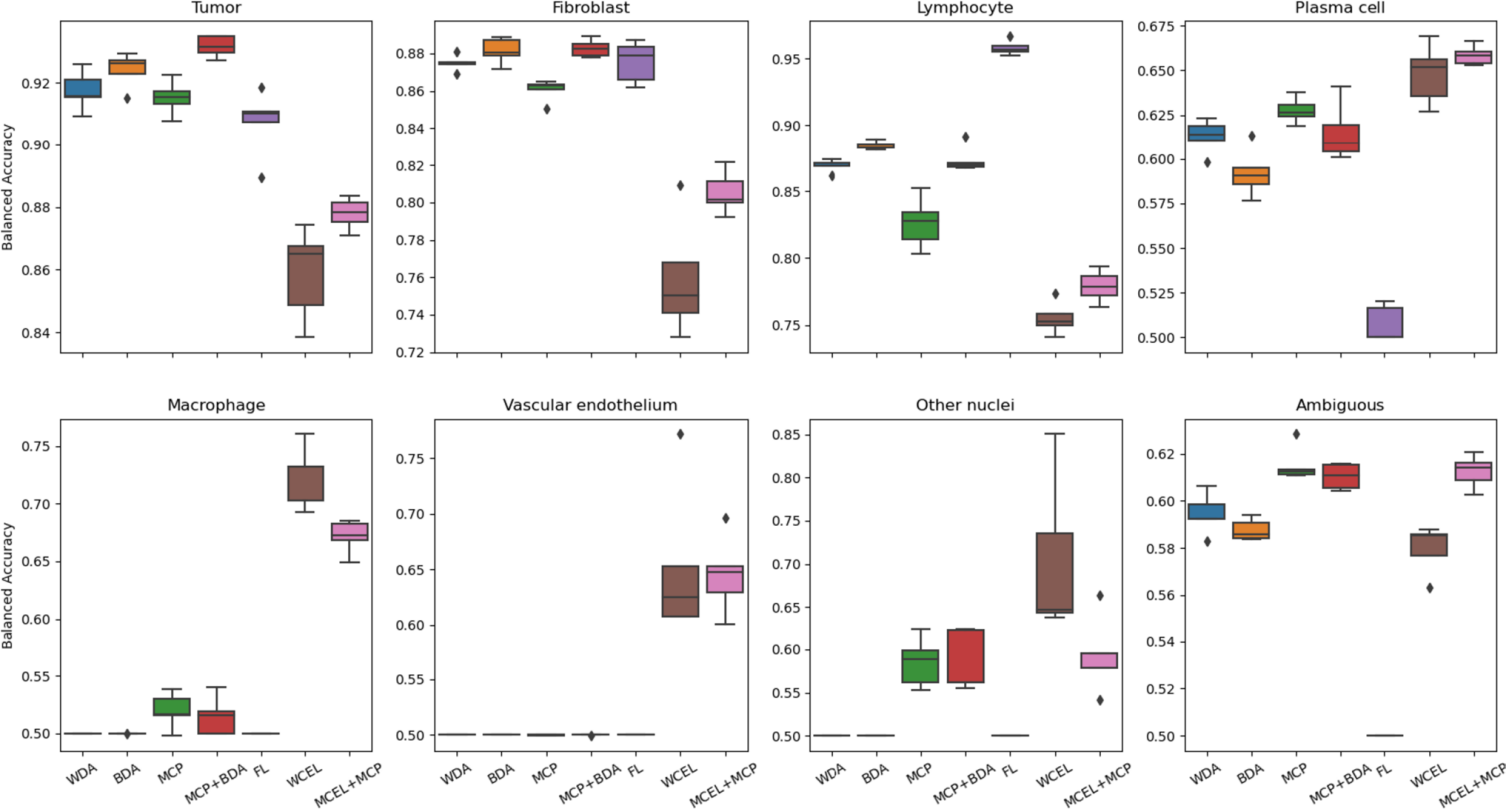
Class-balanced accuracy without considering background label

In our supplementary material, we have included confusion matrices for each experiment. These matrices contain a ‘background’ tag both in the prediction and in the ground truth (GT). On the prediction axis, the ‘background’ tag assists in quantifying the percentage of nuclei that were not detected. Meanwhile, the GT axis helps us determine whether background elements are incorrectly classified as nuclei. By extracting values from these matrices, we calculate the balanced accuracy for each class, considering or disregarding the background label. This information is presented in Figs. 10 and 11, respectively. In summary, Fig. 11 assesses the classification quality of detected nuclei, while Fig. 10 provides a comprehensive evaluation of how well nuclei from each class are both classified and detected. Balanced accuracy combines both sensitivity (true positive rate) and specificity (true negative rate), offering a more comprehensive assessment of each method’s overall classification performance, considering the challenges posed by significant class imbalances.

**Fig. 12:**
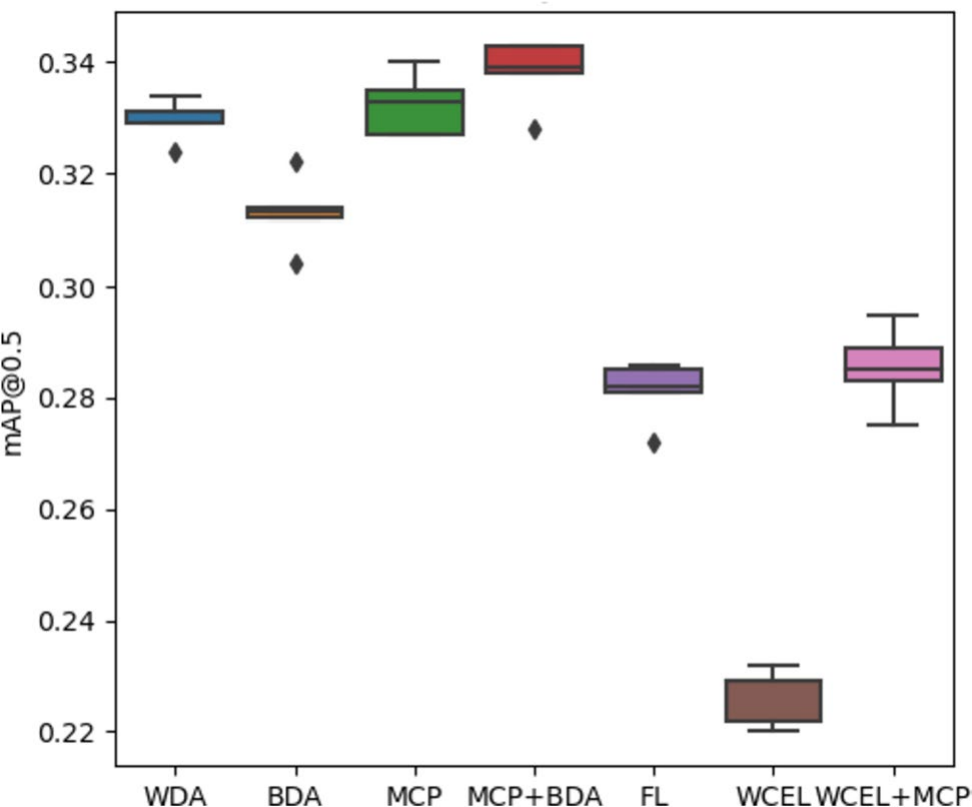
Box plot of mAP@0.5

**Fig. 13:**
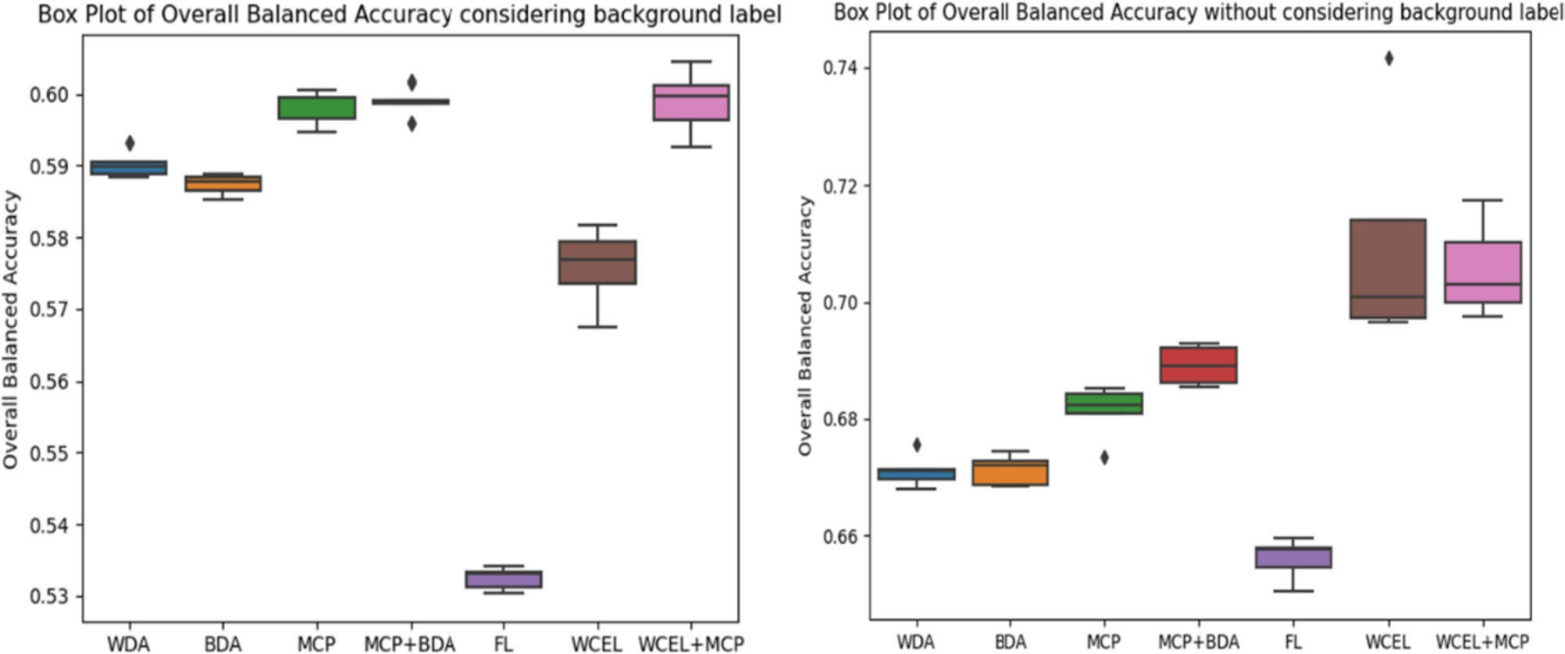
Overall balance accuracy, on the left side, considering the background label and on the right side without considering the background label

The experiments conducted in the second configuration demonstrate that the use of MCP in combination with BDA results in the highest mAP@0.5 score (Fig. 12). However, achieving a higher mAP@0.5 value in highly imbalanced datasets often suggests improved classification primarily in the majority classes, as opposed to all classes, as reflected in Figs. 10 and 11. Therefore, we have used overall balanced accuracy to summarize the performance of each method, which can be observed in Fig. 13.

The findings reveal distinctive patterns in classification performance among different methods. In WDA and BDA, classification performance is notably better for the three major classes (tumor, lymphocyte, and fibroblast), while performance in the remaining classes is poor. This outcome aligns with expectations, given that these experiments do not incorporate methods to address class imbalance.

Contrarily, MCP showcases increased balanced accuracy for minority classes, though certain classes still exhibit subpar sensitivity. When combined with BDA, MCP exhibits similar behavior, albeit with enhanced results in the major classes. Notably, FL demonstrates the poorest performance overall, reducing the detection of majority classes without significantly enhancing the sensitivity of minority classes. The most favorable outcome is observed with the WCEL + MCP combination. In nearly all minority classes, this approach achieves the highest performance without significant degradation in the results of the majority classes.

We utilized the Kruskal–Wallis test to discern variations among independent groups, evaluating the overall balanced accuracy (considering the background label); the resultant *H*-statistic of 31.8857, with a *p*-value of 0.000017, demonstrates significant differences among the groups.

Following the Kruskal–Wallis analysis, a Dunn’s test was conducted using the Bonferroni correction to delve into specific disparities among these groups. The matrix of *p*-values (Table 4) derived from Dunn’s test provides pairwise comparisons between the methods.

**Table 4:** Matrix of *p*-values derived from the Dunn’s test comparing different methods: without data augmentation (WDA), basic data augmentation (BDA), modified copy-paste data augmentation (MCP), modified copy-paste plus basic data augmentation (MCP + BDA), focal loss (FL), weighted cross-entropy loss function (WCEL), weighted cross-entropy plus modified copy-paste data augmentation (WCEL + MCP). The values represent pairwise comparisons, *p*-values below a significance level of 0.05 are highlighted in bold

	**WDA**	**BDA**	**MCP**	**MCP + BDA**	**FL**	**WCEL**	**WCEL + MCP**
**WDA**	1	1	1	1	1	**0.0472**	**0.0345**
**BDA**	1	1	1	1	1	0.0640	**0.0472**
**MCP**	1	1	1	1	0.5096	1	0.8750
**MCP + BDA**	1	1	1	1	**0.0425**	1	1
**FL**	1	1	0.5096	**0.0425**	1	**0.0005**	**0.0003**
**WCEL**	**0.0472**	0.0640	1	1	**0.0005**	1	1
**WCEL + MCP**	**0.0345**	**0.0472**	0.8750	1	**0.0003**	1	1

A key observation from these comparisons is the consistent and substantial differences exhibited by the WCEL + MCP and FL when compared to the other methods. When considering this information in conjunction with Fig. 13, it becomes evident that FL exhibits comparatively lower performance, while WCEL + MCP demonstrates significantly higher performance. These findings suggest statistically significant differences between FL and WCEL + MCP in contrast to the remaining methods.

### Additional Discussion

The current debate surrounding data augmentation techniques in the context of biological imaging, particularly when such techniques distort the global context, is a topic of significant interest. This distortion is evident in algorithms like copy-paste, where instances are copied and pasted into new images, causing a shift in local and global features. One might expect such alterations to lead to a decline in classification performance, as the context surrounding instances is lost. However, our results, especially in the comparison between WDA and MCP in the second dataset configuration, reveal that object prediction in test and validation set images actually improves, indicating superior performance in real-world images.

One explanation for this phenomenon is that by modifying the global context of objects, Convolutional Neural Networks (CNNs) shift their focus toward the extraction of local features rather than global ones. In cases where recurrent patterns are not found in global features, this change in attention to local features can lead to better algorithm performance, as observed in our study.

It should be emphasized that the precise localization and classification of nuclei are intricate tasks that involve identifying very similar elements, which can often be further complicated due to a lack of calibration or focal distance issues, as well as variations in staining within the samples. Additionally, the substantial data imbalance and differences in annotations made by different pathologists contribute to the difficulty in achieving high classification values compared to other object detection studies. However, it is important to recognize that we are continually advancing in the field of complex element detection. These ongoing advances have the potential to become valuable tools for expert pathologists in their work.s

## Conclusions and Future Work

In this study, we introduced a hybrid methodology that effectively addresses the challenges of object detection and classification in highly imbalanced datasets. Our approach combines modified copy-paste data augmentation (MCP) with a weighted loss function, resulting in substantial improvements in the classification of minority classes while maintaining the performance of majority classes. MCP ensures non-overlapping instances and prevents an imbalance between background and foreground, while the weighted loss function optimally distributes attention based on the number of instances. The combination of these methods is strongly recommended for similar tasks.

Our proposed modification to the CP algorithm offers several advantages: (1) *localization evaluation*—it reduces the likelihood of overlapping instances by incorporating a localization evaluation step. (2) C*lass imbalance reduction*—by copying and pasting instances of classes that are not initially present in the image, it helps alleviate class imbalance issues. (3) *Imbalance between background and foreground*—images with low instance densities are more likely to be populated, reducing the risk of creating an imbalance between the background and foreground due to the exclusion of instances that could not find available space.

We plan to combine the NuCLS datasets with the PanNuke dataset to enrich our findings. Collaborating with pathologists will also allow us to augment the samples of minority classes. Furthermore, we aim to extend our experiments by incorporating a Vision Transformer backbone into the Mask RCNN model to explore advanced modeling approaches.

### Supplementary Information

Below is the link to the electronic supplementary material.

Supplementary file1 (DOCX 2510 KB)

## Data Availability

Both NuCLS and Panuke are public datasets, and can be consulted here: https://nucls.grand-challenge.org/ and https://academictorrents.com/details/99f2c7b57b95500711e33f2ee4d14c9fd7c7366c.
