# Supplementary material for: A Data Augmentation Methodology to Reduce the Class Imbalance in Histopathology Images
Source: J Imaging Inform Med. 2024 Mar 14;37(4):1767–82. doi: 10.1007/s10278-024-01018-9 (PMC11300732; doi:10.1007/s10278-024-01018-9)
Supplement: Supplementary file 1 — Supplementary file1 (DOCX 2510 KB) [file 10278_2024_1018_MOESM1_ESM.docx]

Supplementary Data: A data augmentation methodology to reduce the class imbalance in histopathology images.

# Confusing matrices of the second dataset configuration


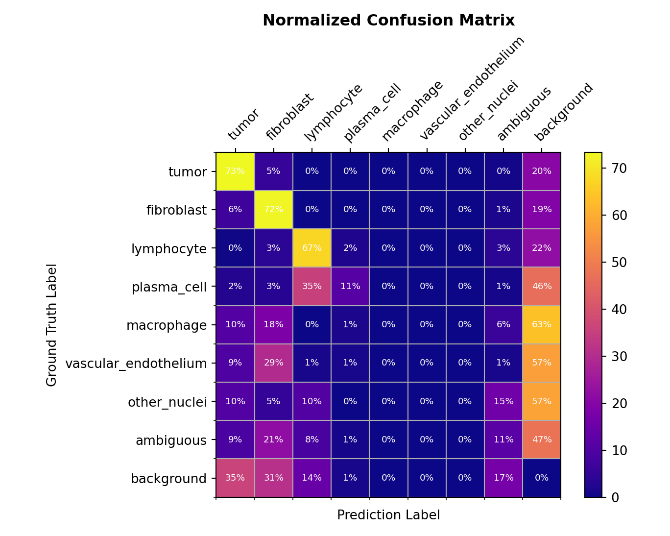

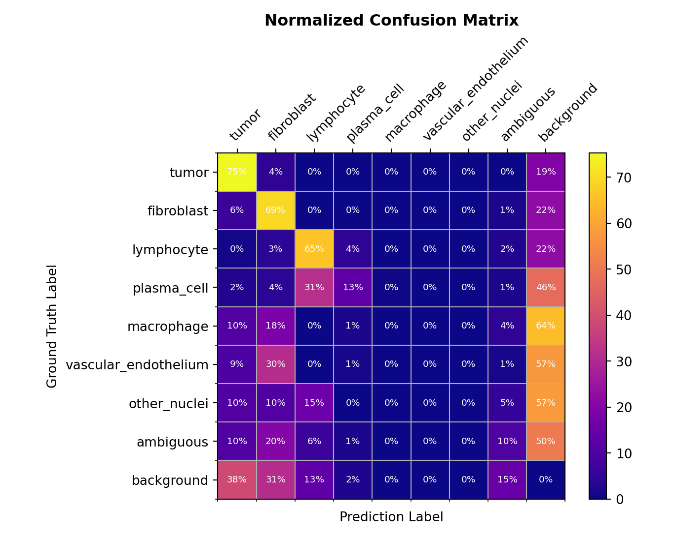

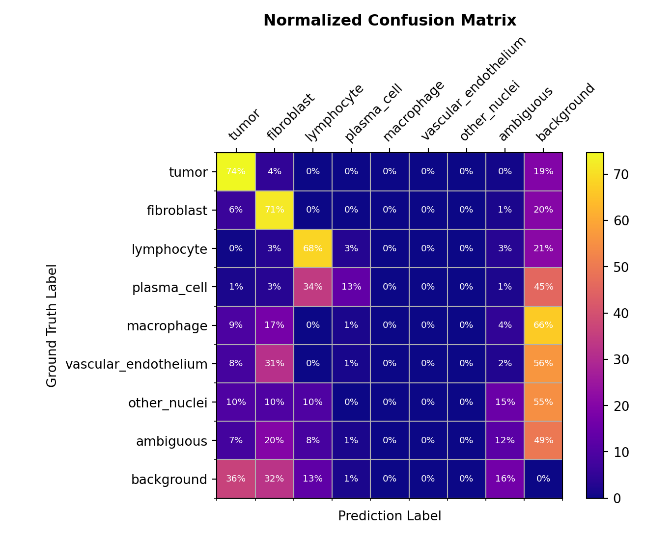

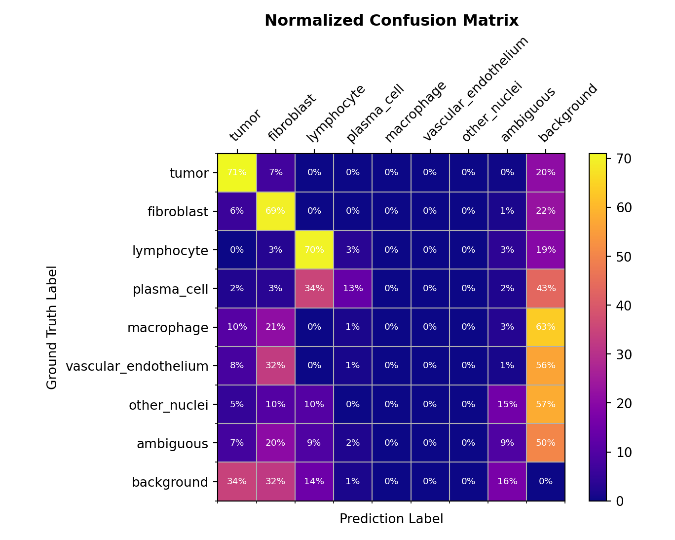

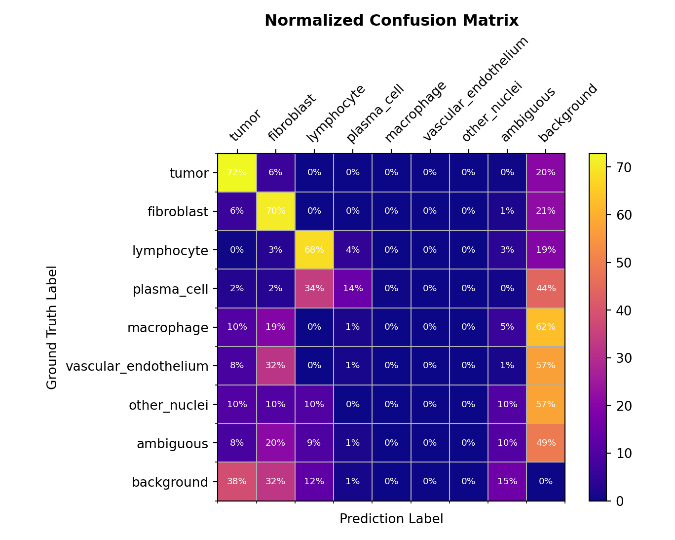


Figure S1. Confusion matrices of WDA using the second dataset configuration. Every matrix corresponds to one subset of the 5-fold cross-validation.


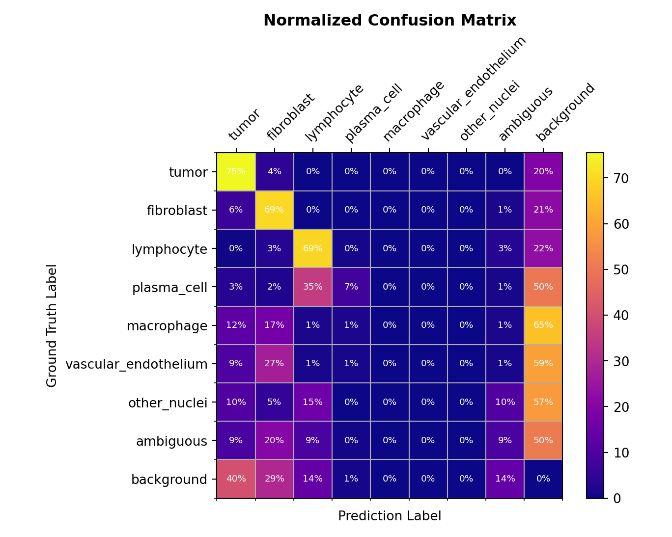

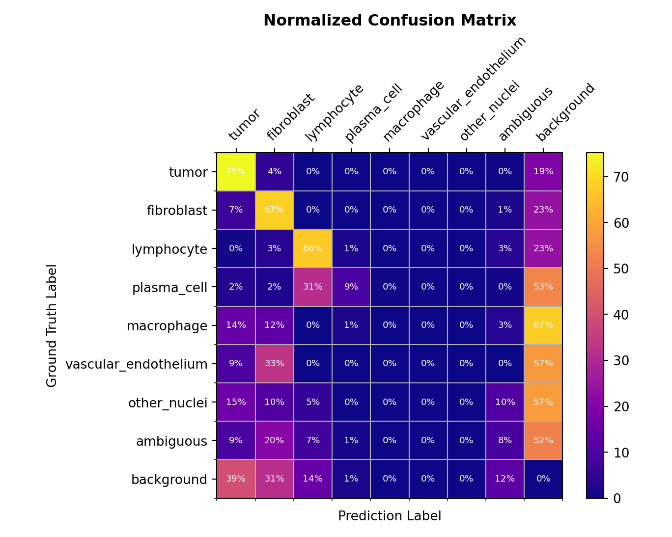

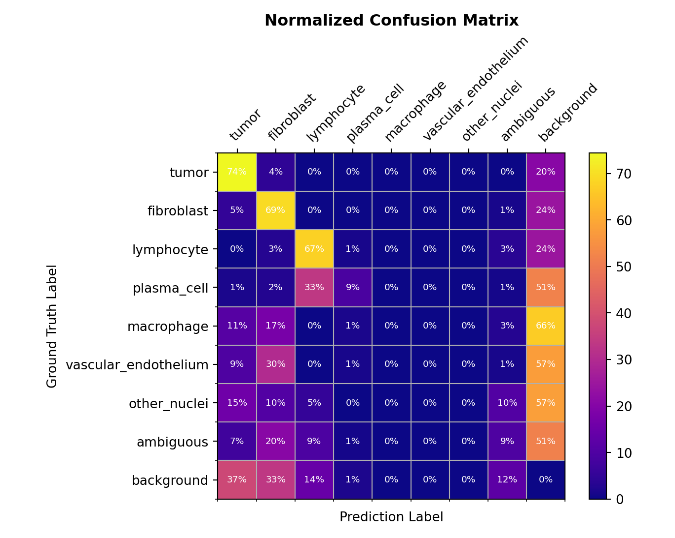

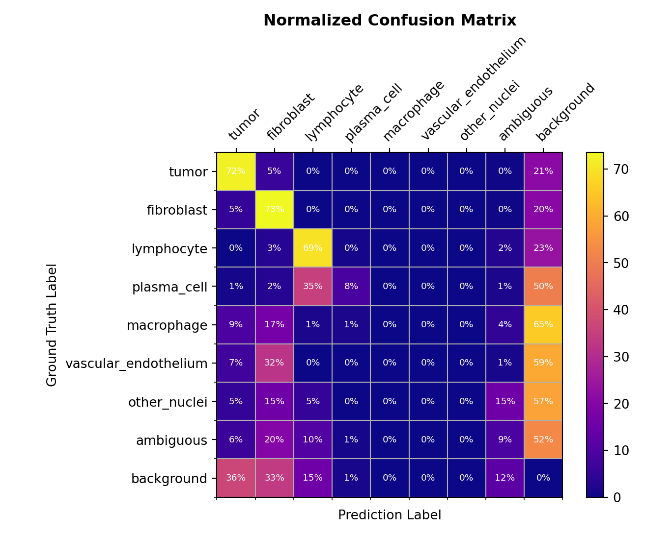

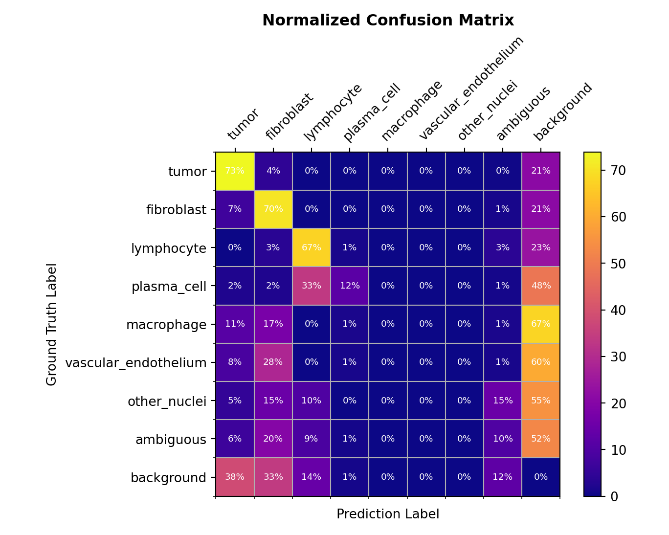


Figure S2. Confusion matrices of BDA using the second dataset configuration. Every matrix corresponds to one subset of the 5-fold cross-validation.


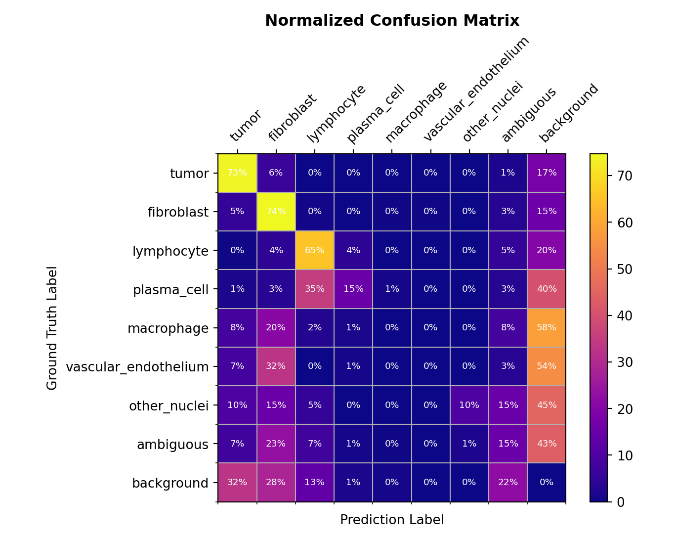

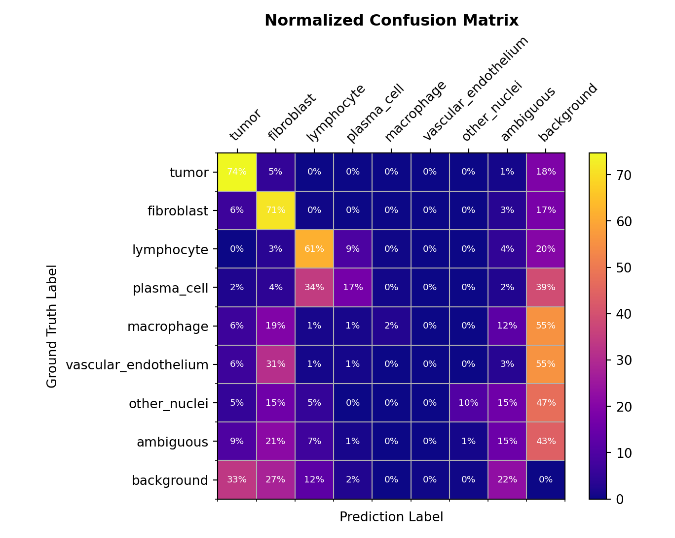

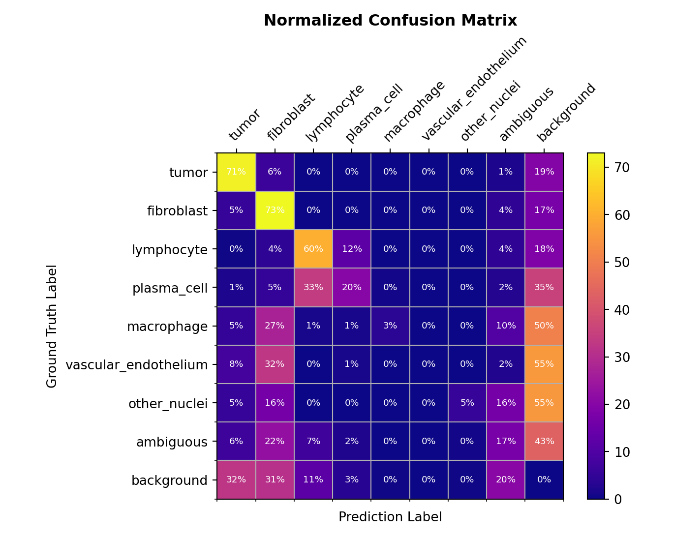

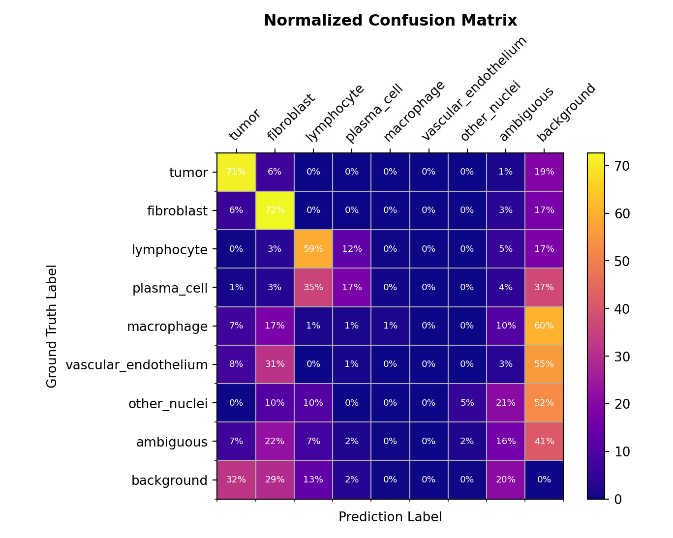

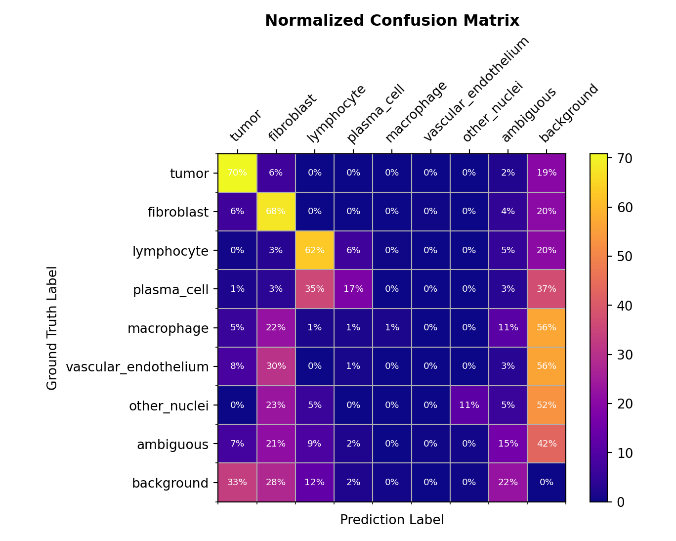


Figure S3. Confusion matrices of MCPP using the second dataset configuration. Every matrix corresponds to one subset of the 5-fold cross-validation.


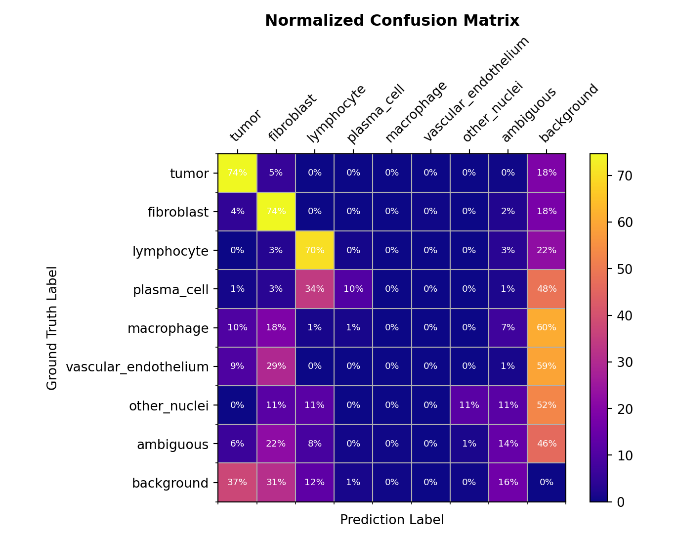

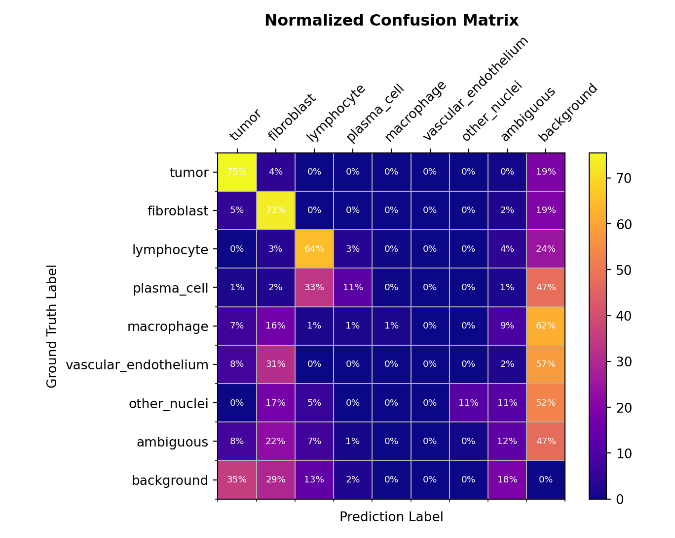

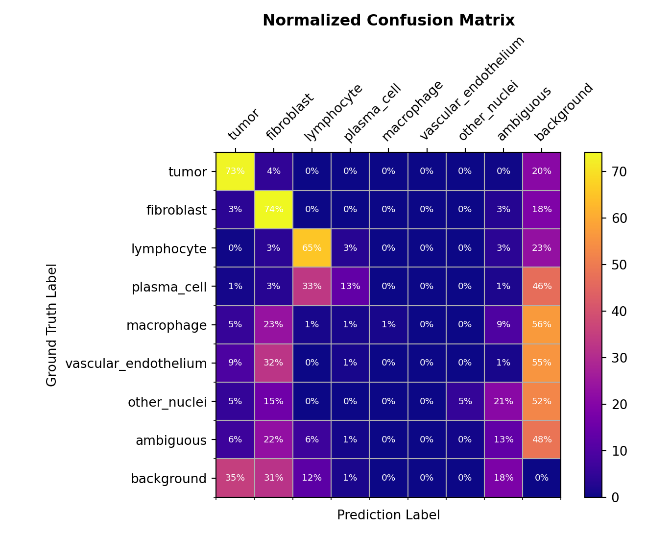

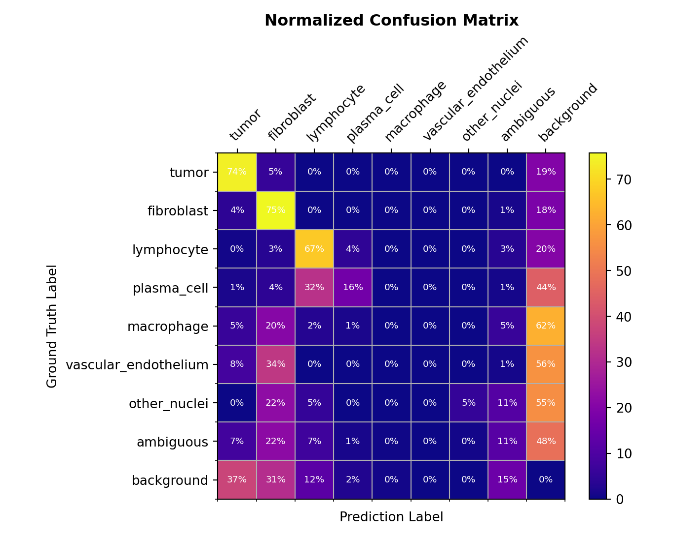

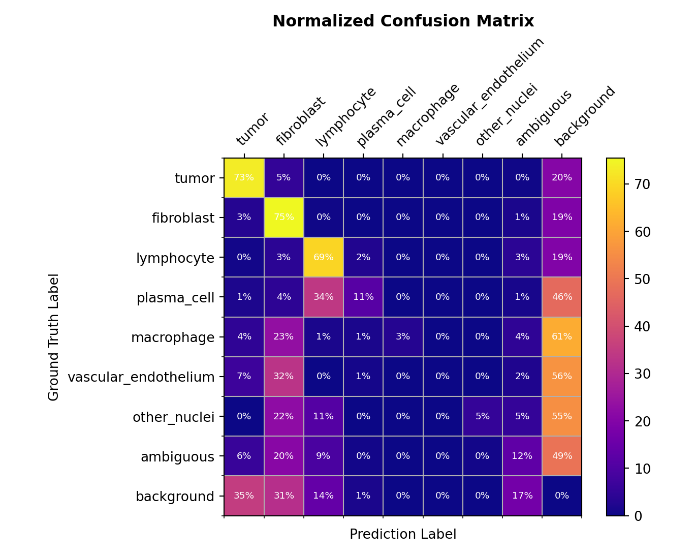


Figure S4. Confusion matrices of MCPP + BDA using the second dataset configuration. Every matrix corresponds to one subset of the 5-fold cross-validation.


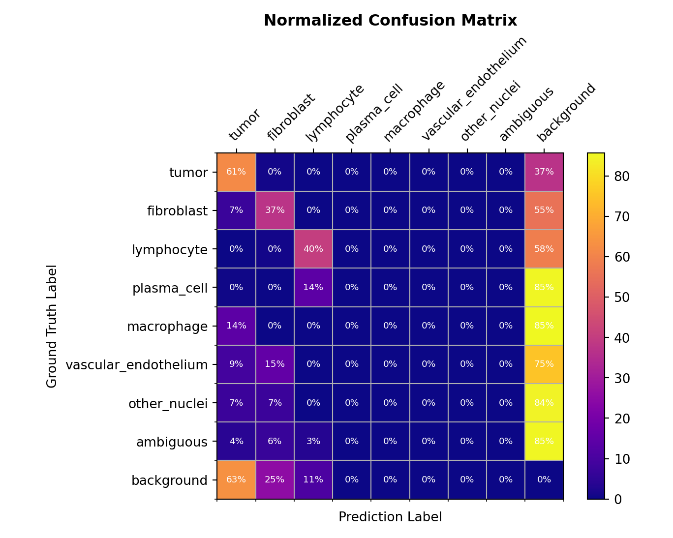

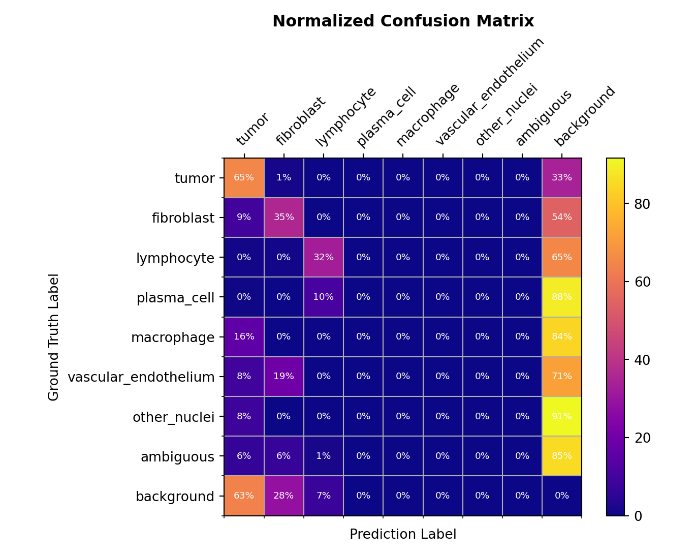

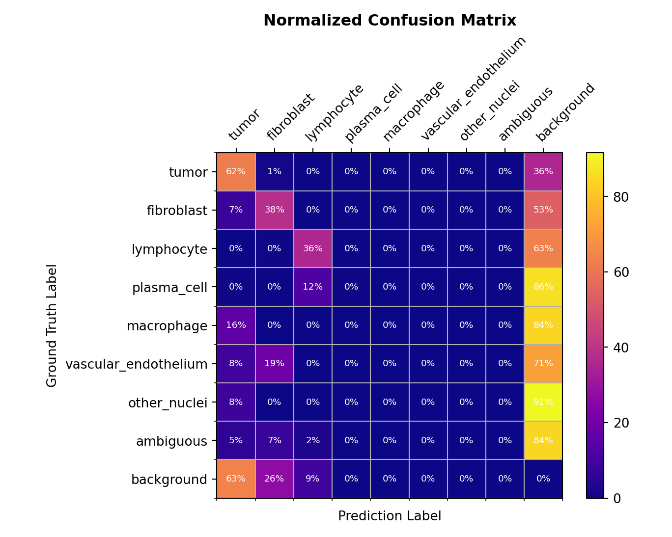

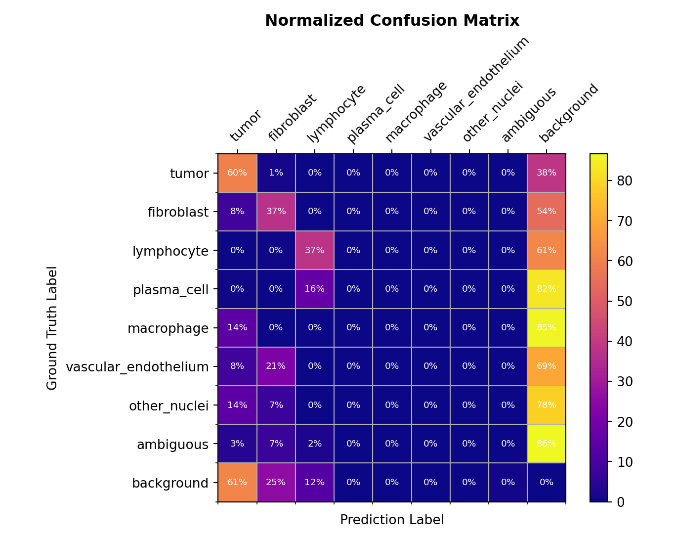

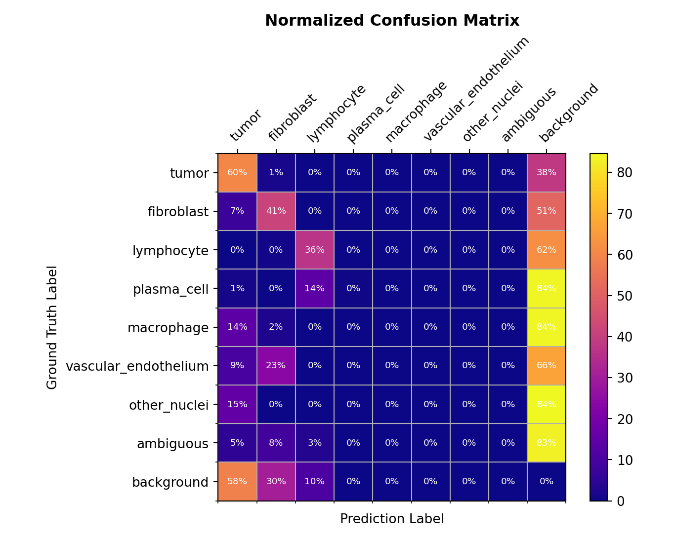


Figure S5. Confusion matrices of FL using the second dataset configuration. Every matrix corresponds to one subset of the 5-fold cross-validation.


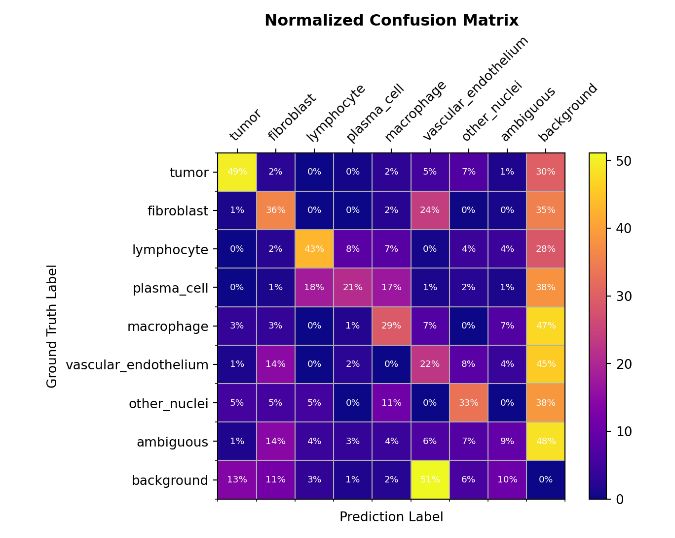

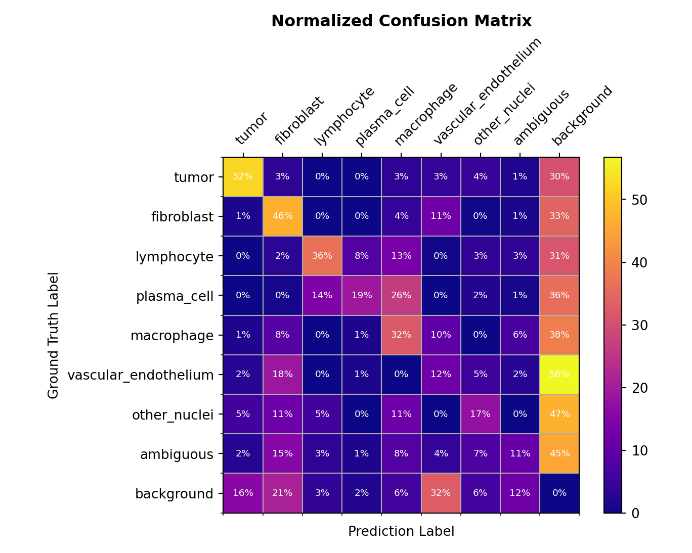

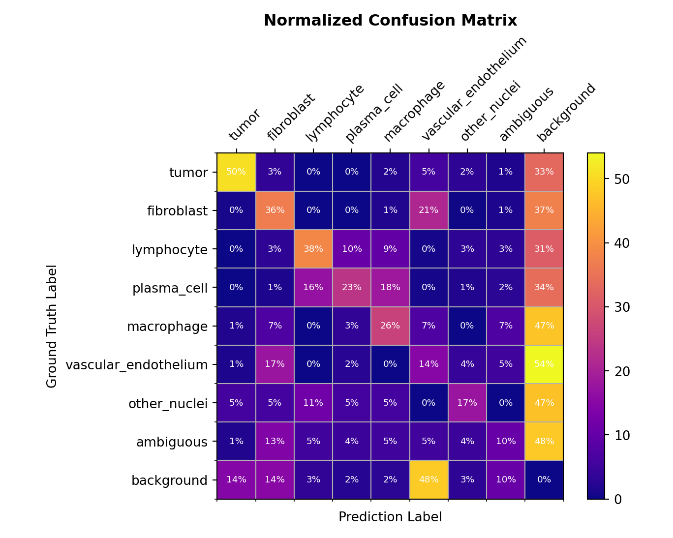

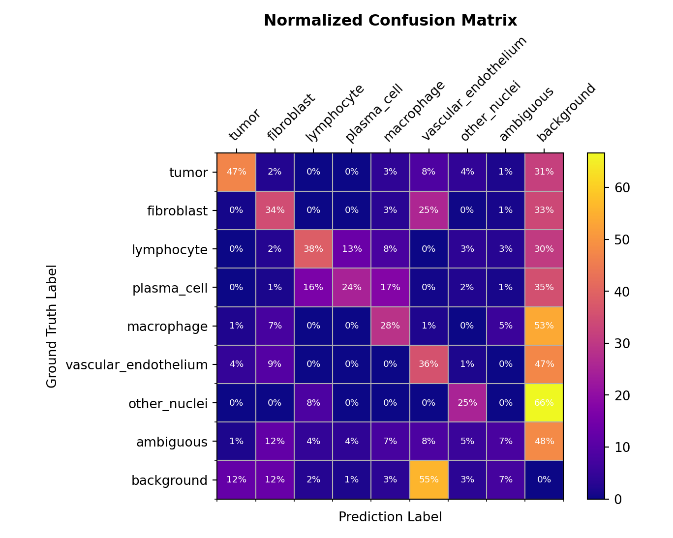


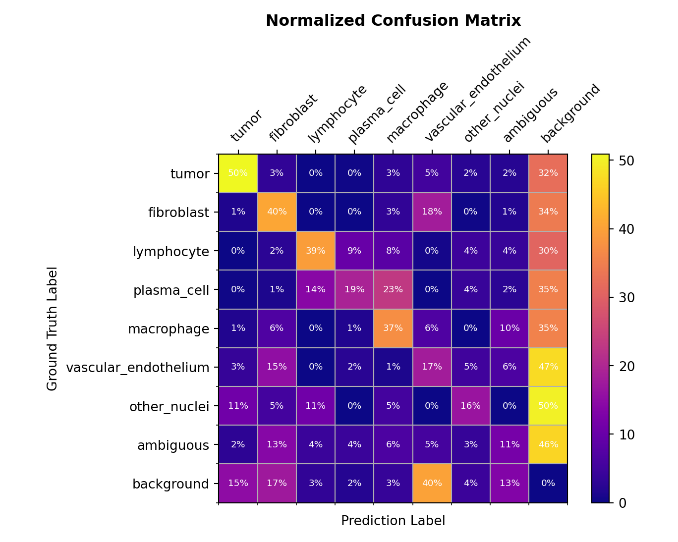


Figure S6. Confusion matrices of WCEL using the second dataset configuration. Every matrix corresponds to one subset of the 5-fold cross-validation.


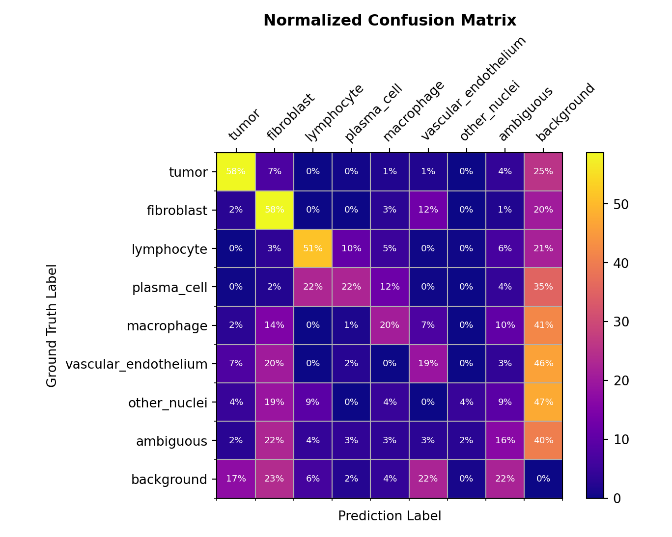

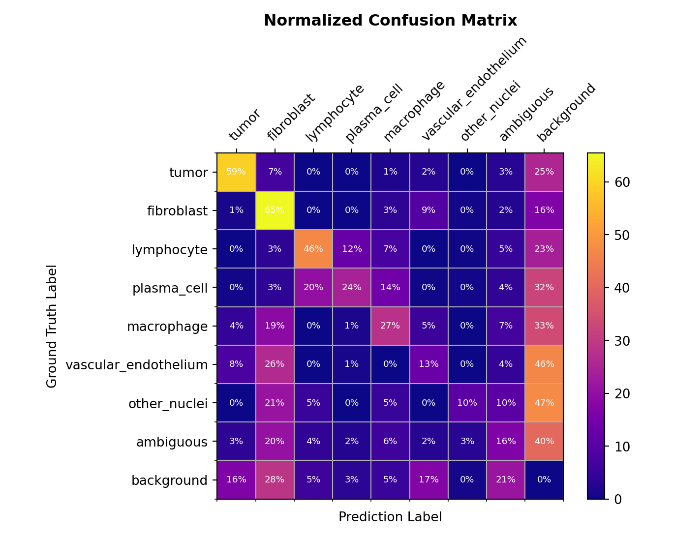

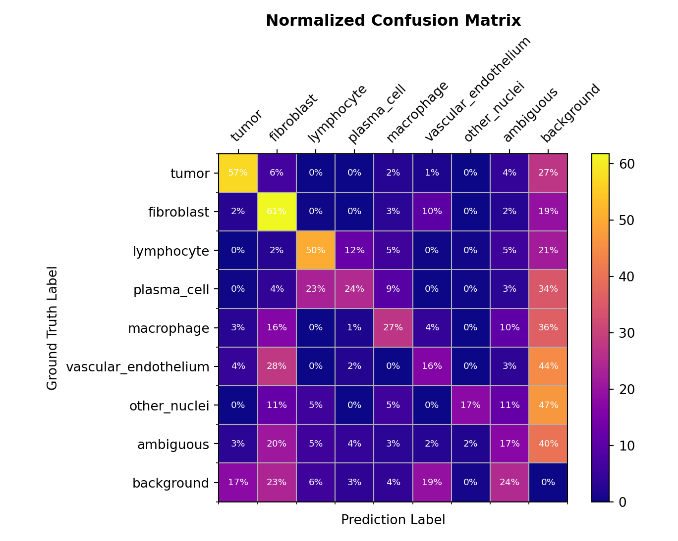

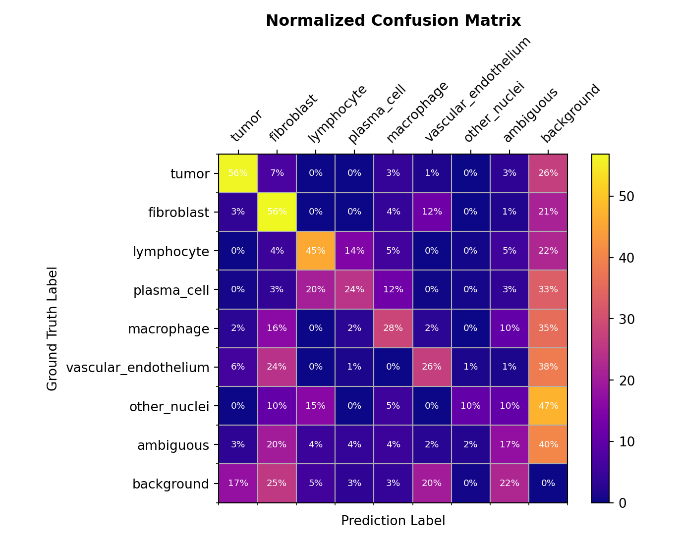


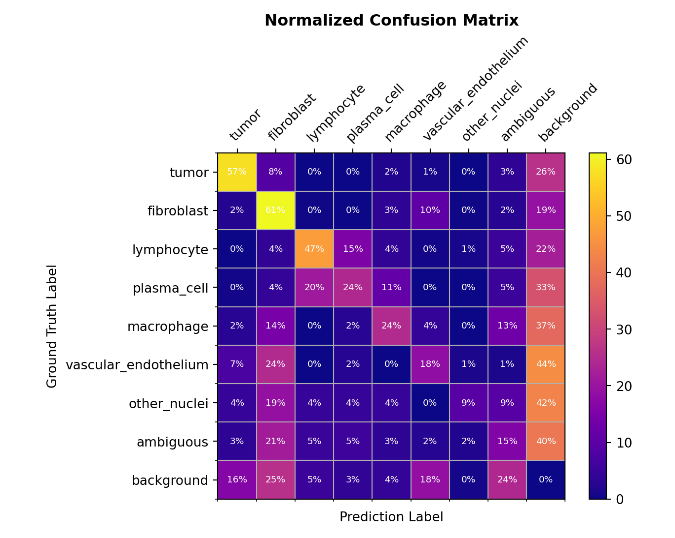


Figure S7. Confusion matrix of WCEL + MCPP using the second dataset configuration. Every matrix corresponds to one subset of the 5-fold cross-validation.
